# Autophagy Regulator Rufy 4 Promotes Osteoclastic Bone Resorption by Orchestrating Cytoskeletal Organization via Its RUN Domain

**DOI:** 10.3390/cells13211766

**Published:** 2024-10-25

**Authors:** Eiko Sakai, Minoru Saito, Yu Koyanagi, Yoshitsugu Takayama, Fatima Farhana, Yu Yamaguchi, Takayuki Tsukuba

**Affiliations:** 1 Department of Dental Pharmacology, Graduate School of Biomedical Sciences, Nagasaki University, 1-7-1 Sakamoto, Nagasaki 852-8588, Japanbb55321204@ms.nagasaki-u.ac.jp (Y.K.); fatimafarhana.2011@gmail.com (F.F.); yu-y@nagasaki-u.ac.jp (Y.Y.); 2 Kondou Dental Clinic, 1154-5 Oozujinnai, Kikuchi 869-1221, Japan; 3 Department of Prosthetic Dentistry, Graduate School of Biomedical Sciences, Nagasaki University, 1-7-1 Sakamoto, Nagasaki 852-8588, Japan; 4 Ito Dental Clinic Medical Corporation, 3-2-4 Kousienn, Nishinomiya 663-8152, Japan

**Keywords:** Rufy4, RUN and FYVE domain-containing protein family, autophagy, osteoclast, bone resorption, cytoskeleton organization, cathepsin K, siRNA, intracellular trafficking

## Abstract

Rufy4, a protein belonging to the RUN and FYVE domain-containing protein family, participates in various cellular processes such as autophagy and intracellular trafficking. However, its role in osteoclast-mediated bone resorption remains uncertain. In this study, we investigated the expression and role of the *Rufy4* gene in osteoclasts using small interfering RNA (siRNA) transfection and gene overexpression systems. Our findings revealed a significant increase in Rufy4 expression during osteoclast differentiation. Silencing *Rufy4* enhanced osteoclast differentiation, intracellular cathepsin K levels, and formation of axial protrusive structures but suppressed bone resorption. Conversely, overexpressing wild-type *Rufy4* in osteoclasts hindered differentiation while promoting podosome formation and bone resorption. Similarly, overexpression of a *Rufy4* variant lacking the RUN domain mimics the effects of *Rufy4* knockdown, significantly increasing intracellular cathepsin K levels, promoting osteoclastogenesis, and elongated axial protrusions formation, yet inhibiting bone resorption. These findings indicate that Rufy4 plays a critical role in osteoclast differentiation and bone resorption by regulating the cytoskeletal organization through its RUN domain. Our study provides new insights into the molecular mechanisms governing osteoclast activity and underscores Rufy4’s potential as a novel therapeutic target for bone disorders characterized by excessive bone resorption.

## 1. Introduction

Intracellular trafficking is vital for various dynamic cellular processes, including structural changes, such as cell migration, fusion, and polarization, as well as the secretion of cellular substances [1,2,3]. Proper coordination between the cytoskeleton and membrane transport is crucial for these processes [4].

The RUN and FYVE domain-containing protein (RUFY) family comprises five relatively conserved genes, namely *Rufy1* to *Rufy4* and *Fyco1*. These genes are associated with membrane trafficking processes, such as endocytosis and autophagy; however, their functions are poorly understood [5]. Rufy4 is a newly identified protein molecule expressed in dendritic cells and alveolar macrophages that plays crucial roles in the formation of autophagosomes and the regulation of macroautophagy [6]. It interacts with Rab7, a protein from the Rab family of small GTPases, through its N-terminal RUN domain, while its C-terminal FYVE domain interacts with phosphatidylinositol 3-phosphate [6]. Mass spectrometry data from HeLa cells stably expressing *Rufy4* revealed that the Rufy4 protein interacts with Rab34 and PLEKHM1, proteins involved in the regulation and fusion of late endosomal membranes [7]. Recent studies using HeLa cells and hippocampal neurons have shown that Rufy4 interacts with Rufy3, acting as an effector of the small GTPase ARL8. This interaction, involving both ARL8 and the dynein-dynactin complex, plays a crucial role in the retrograde transport of endolysosomes along microtubules [8].

Osteoclasts are multinucleated cells derived from the monocyte/macrophage lineage responsible for bone resorption [9]. Upon attachment to the mineralized bone matrix, osteoclasts undergo cytoskeleton reorganization, transforming into highly polarized structures [10]. Specifically, intracellular vesicles migrate toward the bone, fusing with the cell membrane adjacent to the bone to form a ruffled border, a membrane domain, within a region surrounded by a ring-like F-actin-rich podosome known as the sealing zone [11]. Bone resorption through the ruffled border occurs because of the action of mineral-dissolving acids and proteinases, such as cathepsin K, which are secreted toward the bone to degrade bone matrix proteins [12]. Rab7 plays a crucial role in this process by facilitating the transport of polar vesicles and the formation of ruffle borders in osteoclasts [13]. Rho GTPases, including Rho A, Rac, and cdc42, are implicated in regulating podosome assembly and sealing zone formation in osteoclasts [14]. Excessive osteoclast-mediated bone resorption can lead to pathological bone destruction, as seen in conditions such as osteoporosis, rheumatoid arthritis, and alveolar bone resorption in periodontal disease [15].

Osteoclast differentiation is physiologically regulated by the receptor activator of nuclear factor κ-B ligand (RANKL) [16,17], a member of the tumor necrosis factor (TNF) superfamily. Pathologically, it is promoted by several molecules, including inflammatory cytokines, such as TNF-alpha [18] and IL6 [19], lipopolysaccharides [20], and oxidative stress [21]. To combat oxidative stress, the body employs a defense mechanism involving the nuclear factor-erythroid 2-related factor 2- (Nrf2) and Kelch-like ECH-associated protein 1 (Keap1) system [22]. Under normal conditions, Keap1 binds to Nrf2, leading to its proteasomal degradation. However, under oxidative stress conditions, the interaction between Nrf2 and Keap1 is disrupted, enabling Nrf2 to translocate to the nucleus and promote the transcription of phase II antioxidant enzymes, which help mitigate oxidative stress. Our previous study, utilizing splenocytes as osteoclast precursor cells, owing to the juvenile lethality associated with Keap1 deficiency [23], showed significantly enhanced osteoclastic differentiation in *Nrf2*-deficient mice compared to wild-type mice when cultured with RANKL. Conversely, splenocytes from *Keap1*-deficient mice failed to form osteoclasts under the same conditions [24].

In this study, we performed DNA microarray analysis on splenocytes obtained from *Nrf2-* and *Keap1*-deficient mice cultured for 60 h in the presence of RANKL and compared their gene expression profiles. Subsequent meticulous experiments revealed a significant upregulation of *Rufy4* expression in osteoclasts derived from the *Nrf2*-deficient condition. However, the expression and role of *Rufy4* in wild-type osteoclasts remain unclear. Previous studies have indicated that Rufy4 interacts with Rab7 through its RUN domain [6]. Given that Rab7-dependent polarized vesicular trafficking is crucial for the formation of ruffled borders and bone resorption in osteoclasts [13], we hypothesized that Rufy4 regulates bone resorption in osteoclasts through its RUN domain. To elucidate the underlying mechanism, we conducted experiments utilizing small interfering RNA (siRNA) transfection and gene overexpression systems. Deletion of the RUN domain in osteoclasts resulted in extended protrusion formation. Our findings indicate that Rufy4 plays a crucial role in osteoclast differentiation and bone resorption by mediating cytoskeleton organization through its RUN domain, suggesting the potential to develop novel therapeutic strategies for bone diseases.

## 2. Materials and Methods

### 2.1. Reagents and Antibodies

Recombinant RANKL was prepared using an expression plasmid containing the hexahistidine-tagged human soluble RANKL extracellular domain, generously provided by Dr. H. Amano (Tokyo Medical and Dental University, Tokyo, Japan). This process involved a slight modification to a previously described method [25]. The recombinant RANKL was expressed in Codon-Plus BL21 (DE3) RL competent cells and subsequently purified via Ni-NTA column chromatography. Contaminated endotoxins were eliminated through phase separation using Triton X-114 (Nacalai Tesque, Inc., Kyoto, Japan), resulting in a final endotoxin concentration below the detection limit (1 pg/μg protein). The bioactivity of the purified protein was found to be comparable to that of human soluble RANKL (PeproTech EC, London, UK) in an osteoclastogenesis assay performed using mouse bone marrow and RAW-D cells. Osteo Assay Stripwell Plates were purchased from Corning (Corning, NY, USA). Osteoclast precursor cells were isolated from the spleens of newborn mice according to a previously described method [24].

Antibodies (Abs) were purchased from the following sources: Mouse monoclonal anti-Src (# 05-184) was purchased from Upstate Biotechnology (Lake Placid, NY, USA). Rabbit polyclonal anti-β actin (#A5060) and mouse monoclonal anti-FLAG antibody (#F1804, clone M2) were from Sigma Aldrich (St. Louis, MO, USA). The rabbit polyclonal anti-GFP (#598) antibody was purchased from MBL (Nagoya, Japan). Mouse monoclonal anti-NFATc1 (#sc 7294), mouse monoclonal anti-CRIK (#sc390437), and rat monoclonal anti-α-tubulin (#sc 53029) antibodies were purchased from Santa Cruz Biotechnology (Santa Cruz, CA, USA). Rabbit polyclonal antibodies against vinculin (#13901), cortactin (#3502), LIMK (#3842), WASP (#4860), ARP2 (#5614), phospho-cofilin (#3313), cofilin (#5175), RhoA (#2117), Cdc42 (#2462), phospho-Akt (# 4060), Akt (#4691), phospho-JNK (#9251), JNK (#9252), phospho-ERK1/2 (#9101), ERK1/2 (#9102), phospho-p38 MAPK (#4511), p38 MAPK (#9212), and anti-glyceraldehyde-3-phosphate dehydrogenase (GAPDH) (# 2118) antibodies were from Cell Signaling Technology (Danvers, MA, USA). Anti-mouse IgG, HRP-linked antibody (#7076) and anti-rabbit IgG, HRP-linked antibody (#7074), anti-rat IgG (H+L)-Alexa 555 conjugate (#4417), anti-mouse IgG (H+L)-Alexa 555 conjugate (#4409), anti-rabbit IgG (H+L)-Alexa 555 conjugate (#4413), anti-rat IgG (H+L)-Alexa 488 conjugate (#4416), anti-mouse IgG (H+L)-Alexa 488 conjugate (#4408), and anti-rabbit IgG (H+L)-Alexa 488 conjugate (#4412) were purchased from Cell Signaling Technology. Alexa Fluor 488 phalloidin (#A12379) and ProLong Diamond Antifade Mountant with DAPI (#p36962) were obtained from Invitrogen (Carlsbad, CA, USA). A rat monoclonal anti-LAMP1 (CD107a, lysosome-associated membrane protein-1) antibody and anti-LAMP2 (CD107b, lysosome-associated membrane protein-2) were prepared using hybridoma cells kindly provided by Dr. Miki Yokoyama (Tokyo Medical and Dental University, Tokyo, Japan). Briefly, hybridoma cell (1D4B) culture supernatants were collected, and the anti-LAMP1 IgG in the supernatant was eluted using Protein G-sepharose. The IgG fraction was then confirmed by electrophoresis. Mouse monoclonal anti-mDia antibody (#61084) was purchased from BD Biosciences (Franklin Lakes, NJ, USA). Antisera against purified rat spleen cathepsin D [26] and the C-terminal peptide of cathepsin K were prepared as described previously [27].

### 2.2. Animals

Breeding pairs of *Nrf2*^−/−^ and *Keap1* ^+/−^ mice were obtained from the RIKEN Bioresource Center (Tsukuba, Japan) [23,28]. All animal experimental protocols were approved by the Animal Care and Use Committee of the Nagasaki University Graduate School of Biomedical Sciences (Approval Number: 210216169-2).

### 2.3. Cell Culture

For the DNA Microarray, osteoclast precursors obtained from the entire spleen of neonatal mice were utilized, as previously documented [24]. Briefly, spleen cells were cultured in minimal essential medium α (MEMα) supplemented with 10% fetal bovine serum (FBS), penicillin (100 U/mL), streptomycin (100 μg/mL), amphotericin B (0.25 μg/mL), and macrophage-colony stimulating factor (M-CSF; 50 ng/mL) for 24 h at 37 °C under 5% CO_2_. The following day, non-adherent cells were collected and recultured with M-CSF (50 ng/mL). After 72 h, the adherent splenic macrophages were cultured with M-CSF (30 ng/mL) and RANKL (50 ng/mL) to induce their maturation into osteoclasts. For siRNA transfection or gene overexpression, the murine monocytic cell line, RAW-D, kindly provided by Prof. Toshio Kukita (Kyushu University, Japan), was used as a pre-osteoclast model [29,30].

### 2.4. mRNA Expression Profiling During Osteoclast Differentiation by DNA Microarray

To identify genes upregulated during osteoclast differentiation under oxidative stress, we employed the Affymetrix DNA Microarray system (Affymetrix, Santa Clara, CA, USA)**.** Osteoclasts derived from splenocytes of *Keap1*- or *Nrf2*-deficient mice were cultured for 60 h in the presence of M-CSF (30 ng/mL) and RANKL (50 ng/mL) on plastic dishes. Total RNA from these osteoclasts was isolated with TRIzol Reagent (Invitrogen) and subsequently purified with the RNeasy Mini Kit (QIAGEN, Tokyo, Japan) following the manufacturer’s instructions. The hybridized microarray gene chips were analyzed and scanned using an Affymetrix Scanner in accordance with the manufacturer’s instructions.

### 2.5. Quantitative Real-Time Polymerase Chain Reaction (qRT-PCR) Analysis

qRT-PCR analysis was conducted in accordance with previously established protocols [31]. Total RNA was extracted using TRIzol Reagent (Invitrogen) and then reverse transcribed using oligo(dT)15 primer (Promega, Madison, WI, USA) and ReverTra Ace (Toyobo, Osaka, Japan). qRT-PCR was performed using Quant Studio 3 (Thermo Fisher Scientific, Waltham, MA, USA), and cDNA was amplified using Brilliant ΙΙΙ Ultra-Fast SYBR QPCR Master Mix (Agilent Technologies, Santa Clara, CA, USA) following the manufacturer’s instructions. The primer sets utilized for qRT-PCR were as follows.

*Gapdh*, forward: 5′-AAATGGTGAAGGTCGGTGTG-3′ and reverse: 5′-TGAAGGGGTCGTTGATGG-3′; *β-actin*, forward: 5′-ACCCAGATCATGTTTGAGAC-3′ and reverse: 5′-GTCAGGATCTTCATGAGGTAGT-3′; *Rufy4*, forward: 5′-AGGGACGCCATGTATCAGAC-3′ and reverse: 5′-GAGACAGACGCCTGAAGACC-3′; *CtsK*, forward: 5′-CAGCTTCCCCAAGATGTGAT-3′ and reverse: 5′-AGCACCAACGAGAGGAGAAA-3′; *Ocstamp*, forward: 5′-TGGGCCTCCATATGACCTCGAGTAG-3′ and reverse: 5′-TCAAAGGCTTGTAAATTGGAGGAGT-3′; *Dcstamp*, forward: 5′-CTAGCTGGCTGGACTTCATCC-3′ and reverse: 5′-TCATGCTGTCTAGGAGACCTC-3′; *Src*, forward: 5′-AGAGTGCTGAGCGACCTGTGT-3′ and reverse: 5′-GCAGAGATGCTGCCTTGGTT-3′; *Nfatc1*, forward: 5′-TCATCCTGTCCAACACCAAA-3′ and reverse: 5′-TCACCCTGGTGTTCTTCCTC-3′; *RelA*, forward: 5′-GCGTACACATTCTGGGGAGT-3′ and reverse: 5′-GTTAATGCTCCTGCGAAAGC-3′; Rab7, forward: 5′-GGCCTTCTACAGAGGTGCAG-3′ and reverse: 5′-TCTTTGTGGCCACTTGTCTG-3′; *Lamp1*, forward: 5′-ATGGCCAGCTTCTCTGCCTCC-3′ and reverse: 5′-ACAGTGGGGTTTGTGGGCAC-3′; *Calcr*, forward: 5′-CGCATCCGCTTGAATGTG-3′ and reverse: 5′-TCTGTCTTTCCCCAGGAAATGA-3′; *Acp5*, forward: 5′-CAGCTGTCCTGGCTCAAAA-3′ and reverse: 5′-ACATAGCCCACACCGTTCTC-3′; *CtsD*, forward: 5′-CTGCTGGGTCCACCATAAGT-3′ and reverse: 5′-CTTGGCTGCAACAAATACGA-3′.

### 2.6. TRAP Staining and Bone Resorption Assay

Cells were fixed with 4% paraformaldehyde (PFA) for 1 h on ice and stained for tartrate-resistant acid phosphatase (TRAP) activity using a previously described method [32]. Mature osteoclasts were identified as TRAP-positive cells with three or more nuclei. The bone resorption assay was performed as outlined in a previous study [33]. In this assay, RAW-D cells were seeded on Osteo Assay Striptwell Plates and cultured with RANKL (500 ng/mL) for 7–10 days. Every 72 h, half of the culture medium was replaced with equal amounts of MEMα containing the same concentrations of RANKL and FBS. After 7–10 days, cells were resuspended in 5% sodium hypochlorite. Images of the resorption areas were captured using a reverse-phase microscope (Plympus CKX41; Tokyo, Japan). To quantify the bone resorption area, the ratio of the resorbed areas to the total areas per image was calculated using the Image J software (Image J 1.53e, National Institutes of Health, Bethesda, MD, USA) (http://rsbweb.nih.gov/ij/, accessed on 17 October 2024).

### 2.7. Small Interfering RNA (siRNA)

siRNA transfection of RAW-D cells was performed as previously described [34]. The target sequences of murine *Rufy4* siRNA were as follows: 5′-GAGUUGCCUUCAACUUGGACCUGCA-3′ (mouse *Rufy4* siRNA #1), 5′-GGAGGGUGUUGGAACUGAUCCAUGA-3′ (mouse *Rufy4* siRNA #2), and 5′-GACAGAAGAUCAGAGAACAACAGAA-3′ (mouse *Rufy4* siRNA #3) (Stealth siRNA; Invitrogen). Briefly, 10 pmol of one of the three independent duplex siRNAs covering the above target sequences or 10 pmol of non-targeting siControl were transfected into RAW-D cells using Lipofectamine RNAiMAX transfection reagent (Invitrogen) according to the manufacturer’s instructions. The cells were then incubated for 24 h. The following day, these cells were transfected again with the siRNA and incubated for an additional 24 or 48 h.

### 2.8. Western Blot Analysis

Western blot analysis was performed as previously described [35]. Cells were rinsed twice with ice-cold phosphate-buffered saline (PBS) and lysed in RIPA buffer [50 mM Tris-HCl (pH 8.0), 1% Nonidet P-40, 0.5% sodium deoxycholate, 0.1% sodium dodecyl sulfate (SDS), 150 mM NaCl] supplemented with a proteinase inhibitor cocktail (Sigma-Aldrich, St. Louis, MO, USA) and 2 mM phenylmethylsulfonyl fluoride (PMSF). Subsequently, the cell lysates were centrifuged at 15,000× *g* for 5 min. The protein concentration of each cell lysate was determined using the Pierce^™^ BCA Protein Assay kit (Thermo Scientific, Rockford, IL, USA), according to the manufacturer’s instructions. Equal amounts of protein were separated by electrophoresis on a 10% SDS-polyacrylamide gel and transferred onto polyvinylidene fluoride (PVDF) membranes. The blots were blocked in Tris-buffered saline containing 3% skim milk and 0.1% Tween 20, followed by incubation with specific antibodies at 4 °C for 12 h. After washing, the blots were incubated with horseradish peroxidase-conjugated secondary antibodies (Cell Signaling Technology) and then probed with Immobilon^®^ Forte Western HRP Substrate (Millipore, Burlington, MA, USA). Immunoreactive bands were detected by an image analyzer (LAS4000 mini, Fuji Film, Tokyo, Japan). Image J software (Image J 1.53e, National Institutes of Health, Bethesda, MD, USA) was used to quantify the digital data of the optical density.

### 2.9. Immunocytochemistry

Immunocytochemistry was performed according to previously established protocols [36]. Briefly, cells were seeded and grown on glass coverslips in the presence of RANKL (100 ng/mL). After 3 days, the cells were fixed with 4% paraformaldehyde in PBS for 30 min on ice and then permeabilized with 0.2% Triton X-100 in PBS for 5 min at 25 °C. The cells were blocked with 0.2% gelatin for 1 h, followed by overnight incubation at 4 °C with primary antibodies against tubulin or LAMP1. After washing the cells thrice with PBS, they were stained with anti-rat IgG-Alexa Fluor 555 conjugate as a secondary antibody and Alexa Fluor 488 phalloidin to visualize F-actin. The samples were visualized using a laser scanning confocal imaging system (LSM800; Carl Zeiss, AG, Jena, Germany).

### 2.10. Retrovirus Construction and Rufy4 Overexpression

Retrovirus construction for overexpression experiments was carried out according to previously described methods [37]. Full-length mouse *Rufy4* cDNA (NM_001170641.1) was amplified by PCR with PrimeSTAR GXL DNA polymerase (Takara, Tokyo, Japan) using cDNA derived from RAW-D cells stimulated with RANKL for 72 h as the template. The following primers were used for the PCR amplification of full-length *mRufy4*: 5′-GACGAGCTGTACAAGGCCAACAAC-3′ (eGFP-Rufy4, forward primer) and 5′-TACCCGGTAGAATTCTCAGGTGTCCTGGATTTC-3′ (vector-EcoRI-mRufy4, reverse primer). To generate the eGFP-Rufy4 fusion protein, the amplified fragments were cloned into a linearized eGFP-tagged pMSCVpuro vector, kindly provided by Prof. Kosei Ito (Nagasaki University, Japan), using the InFusion Cloning Kit (Clontech, Mountain View, CA, USA), with eGFP-pMSCVpuro serving as the control vector.

A 1×FLAG tagged-pMSCVpuro vector was constructed using synthetic oligonucleotides: 5′-CCGGAATTAGATCTCTCGAGATGGACTACAAGGACGACGATGATAAGGAATTCTACCGGGTAGGGGAG-3′ (vector with XhoI and 1x FLAG-vector with EcoRI). To assemble the vector, a forward primer with an XhoI site (5′-CCGGAATTAGATCTCTCGAGATGG-3′) and reverse primer with an EcoRI site (5′-CTCCCCTACCCGGTAGAATTC-3′) were employed. The linearized pMSCVpuro vector, with the eGFP region eliminated by XhoI and EcoR1, was used for the InFusion reaction. To generate the 1×FLAG-Rufy4 fusion protein, the amplified fragments were cloned into the linearized 1×FLAG-tagged pMSCVpuro vector using the InFusion Cloning Kit, with 1×FLAG-tagged pMSCVpuro as the control vector.

The *Rufy4* RUN-domain deletion mutant cDNAs were synthesized by PCR using specific primers for the synthesis of 1xFLAG-Rufy4 (1–200): forward, 5′-GACGACGATGATAAGGCCAACAACGGGACCATC-3′ and reverse, 5′-CAGGTCTGGCCGCTGGTGTAGCTCAGC-3′. Similarly, the primers used for the synthesis of *Rufy4* (490–1692) were as follows: forward, 5′-CAGCGGCCAGACCTGGATGAAGCC-3′, and reverse, 5′-TACCCGGTAGAATTCTCAGGTGTCCTGGATTTC-3′. To produce the 1×FLAG-Rufy4 fusion protein with a deleted RUN-domain, an InFusion reaction was carried out using the aforementioned PCR fragments and the linearized FLAG-tagged pMSCVpuro. Constructs for eGFP-alone, eGFP-Rufy4, 1×FLAG-alone, 1×FLAG-Rufy4, and 1×FLAG-Rufy4 RUN-domain deletion mutant vector were transfected into HEK293T cells using Lipofectamine 3000 (Life Technologies, Carlsbad, MD, USA), according to the manufacturer’s instructions. After 48 h of incubation at 37 °C in 5% CO_2_, supernatants containing the viruses were harvested and used to infect RAW-D cells. Following viral infection, cells were selected using puromycin (5 μg/mL) in MEMα containing 10% FBS, with fresh medium replaced every 3 days. After two weeks of culture, puromycin-resistant cloned cells were obtained.

### 2.11. In Vitro Live-Cell Imaging

RAW-D cells were grown on glass-bottom culture dishes (35 mm CELLview Dish, Greiner Bio-one, Tokyo, Japan) in the presence of RANKL (100 ng/mL) for 48 h. Thereafter, the movement of osteoclasts was recorded under phase contrast every 6 min for 12 h using an all-in-one microscope (BZ-X800, KEYENCE, Osaka, Japan) equipped with a 22.2× lens, a time-lapse module (BZ-H4XT, KEYENCE, Osaka, Japan), a stage-top chamber, and a temperature controller with a built-in CO_2_ gas mixer (INUG2-KIW, Tokai hit, Shizuoka, Japan) at 37 °C and 5% CO_2_. These images were then compiled into video clips.

### 2.12. Detection of Activated Small GTPases

Small GTPases activation assay was performed using the RhoA/ Rac1/ Cdc42 Activation Assay Combo kit (Cell Biolabs, Inc. SanDiego, CA, USA) according to the manufacturer’s protocol. Briefly, cells were cultured in 60 mm culture dishes in the presence of RANKL (100 ng/mL). After 3 days, the cells were washed twice with ice-cold PBS and lysed in lysis buffer supplemented with a proteinase inhibitor cocktail and PMSF. Subsequently, cell lysates were centrifuged at 14,000× *g* for 10 min. The protein concentration of each cell lysate was determined using the Pierce^™^ BCA Protein Assay kit (Thermo Scientific, Rockford, IL, USA), according to the manufacturer’s instructions. Equal amounts of protein (800 μg) were used in the small GTPase pull-down assay. Cell lysates were incubated with Rhotekin RBD beads. The pulled-down proteins were processed for western blotting using an anti-RhoA antibody. Cell lysates were also incubated with GTPγS and GDP as positive and negative controls, respectively.

### 2.13. Cell Viability Assay

Cell viability was measured using a Cell Counting Kit-8 (CCK-8; Dojindo, Kumamoto, Japan). The cells were cultured with RANKL (100 ng/mL). After 3 days, the cells were incubated with CCK-8 for 2 h, and the absorbance at 450 nm was measured.

### 2.14. Morphological Analysis

To analyze cell shape, cells were stained with Alexa Fluor 488 phalloidin and DAPI to visualize F-actin and nuclei. Microscopic images were captured using a laser-scanning confocal imaging system (LSM800). Using these images, cellular boundaries were traced and categorized according to their contour as follows: (Type 1, traced by blue) curves without large bumps and protrusions, (Type 2, traced by yellow) curves with hair-like structures, and (Type 3, traced by red) protrusive complex curves with sharp structures more like a needle than a hair. The length of each contour interval was measured using Image J software (Image J 1.53e, National Institutes of Health, Bethesda, MD, USA). The percentage of total type 1 border length or the percentage of total type 2 plus type 3 border length to total cell perimeter length was calculated.

### 2.15. Statistical Analysis

All data are presented as mean value ± standard deviation (SD) from at least three independent experiments. Statistical significance was determined using a two-tailed Student’s *t*-test for comparison between two groups or the Tukey-Kramer method for comparisons among three or more groups. Differences were considered statistically significant at * *p* < 0.05 and ** *p* < 0.01.

## 3. Results

### 3.1. Rufy4 Expression Increases During RANKL-Stimulated RAW-D Cell Differentiation into Osteoclasts

**Figure 1:**
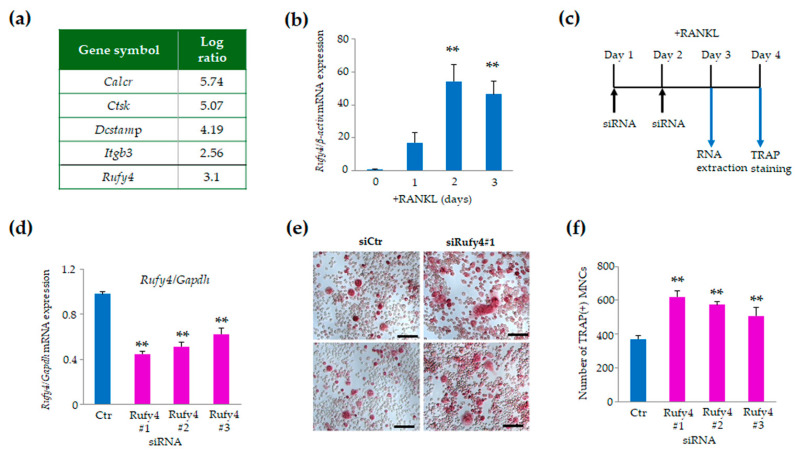
Increased *Rufy4* expression during osteoclast differentiation and impaired osteoclast formation following *Rufy4* knockdown. (**a**) List of upregulated transcripts in *Nrf2*-deficient osteoclasts compared to *Keap1*-deficient cells. (**b**) *Rufy4* mRNA expression was assessed by quantitative real-time PCR during osteoclast differentiation in RAW-D cells treated with 100 ng/mL RANKL. Data are presented as the mean ± SD from 3 independent experiments (** *p* < 0.01). (**c**) Schematic representation of the *Rufy4* knockdown experiment schedule. (**d**) *Rufy4* knockdown efficacy was assessed by measuring the *Rufy4* mRNA levels. RAW-D cells were transfected with control or Rufy4-specific siRNA probes on days 1 and 2 in the presence of 100 ng/mL RANKL. Cells were collected on day 3, and RNA was prepared. Data are presented as the mean ± SD from 3 independent experiments (** *p* < 0.01). (**e**) Tartrate-resistant acid phosphatase (TRAP) staining of control and *Rufy4*-knockdown osteoclasts. Control or Rufy4-knockdown RAW-D cells were stimulated with RANKL (100 ng/mL). On day 4, cells were fixed and stained with TRAP. Scale bar, 50 μm. (**f**) The number of TRAP-positive multinucleated cells was counted. Data are presented as the mean ± SD from 3 independent experiments (** *p* < 0.01).

To identify novel molecules influencing osteoclast differentiation under oxidative stress conditions, we isolated splenocytes from the spleens of 1-day-old wild-type, *Keap1*- [23] and *Nrf2*-deficient mice [28], considering the premature mortality of Keap1-deficient mice [24]. Following a 60-h culture period with M-CSF and RANKL and subsequent DNA microarray analysis on the resulting RNA, we aimed to identify any differences in gene expression patterns that could elucidate the mechanisms underlying osteoclast formation. Previously, we demonstrated that under these culture conditions, osteoclast formation was significantly increased in *Nrf2*-deficient cells compared to wild-type cells, whereas no osteoclasts were observed in *Keap1*-deficient cells after a 60-h culture period. Of the 34 390 genes identified during the analysis, 726 were significantly upregulated more than 2-fold in *Nrf2*-deficient osteoclasts compared to *Keap1*-deficient cells. Notably, osteoclast marker genes, such as calcitonin receptor (*Calcr*), cathepsin K (*Ctsk*), transmembrane 7 superfamily member 4 (*Dcstamp*), and integrin β3 (*Itgb3*), were upregulated. Among these 726 upregulated genes, we focused our attention on *Rufy4*, which showed more than 8-fold upregulation compared to other genes (Figure 1a). Subsequently, we evaluated Rufy4 expression levels in RANKL-stimulated RAW-D cells using qRT-PCR. The results revealed a significant upregulation of *Rufy4* mRNA expression in RANKL-stimulated RAW-D cells at 2- and 3-days post-stimulation, compared to unstimulated RAW-D cells (Figure 1b). These findings suggest that *Rufy4* expression is significantly elevated during RANKL-stimulated differentiation of RAW-D cells into osteoclasts, highlighting Rufy4 as a potentially crucial factor in osteoclastogenesis.

### 3.2. Rufy4 Knockdown Promotes Osteoclast Formation but Impairs Resorption

To investigate the role of *Rufy4* in osteoclast differentiation during osteoclastogenesis, we conducted knockdown experiments by transfecting RANKL-stimulated RAW-D cells with three distinct siRNAs for two consecutive days (Figure 1c). Transfection with siRNA #1 resulted in an approximately 60% decrease in *Rufy4* levels in the cells, whereas siRNA #2 and #3 induced reductions of approximately 50% and 40%, respectively, compared to the control siRNA (Figure 1d). Under these culture conditions with RANKL (100 ng/mL), *Rufy4*-depleted cells exhibited increased osteoclast formation compared to the control cells (Figure 1e). Furthermore, the number of tartrate-resistant acid phosphatase (TRAP)-positive multinucleated cells (MNCs) was significantly higher in *Rufy4*-knockdown cells than in the control cells on day 4 (Figure 1f). Consequently, siRNA #1 was selected for further knockdown experiments owing to its highest knockdown efficiency and consistent osteoclastogenesis inhibition.

**Figure 2:**
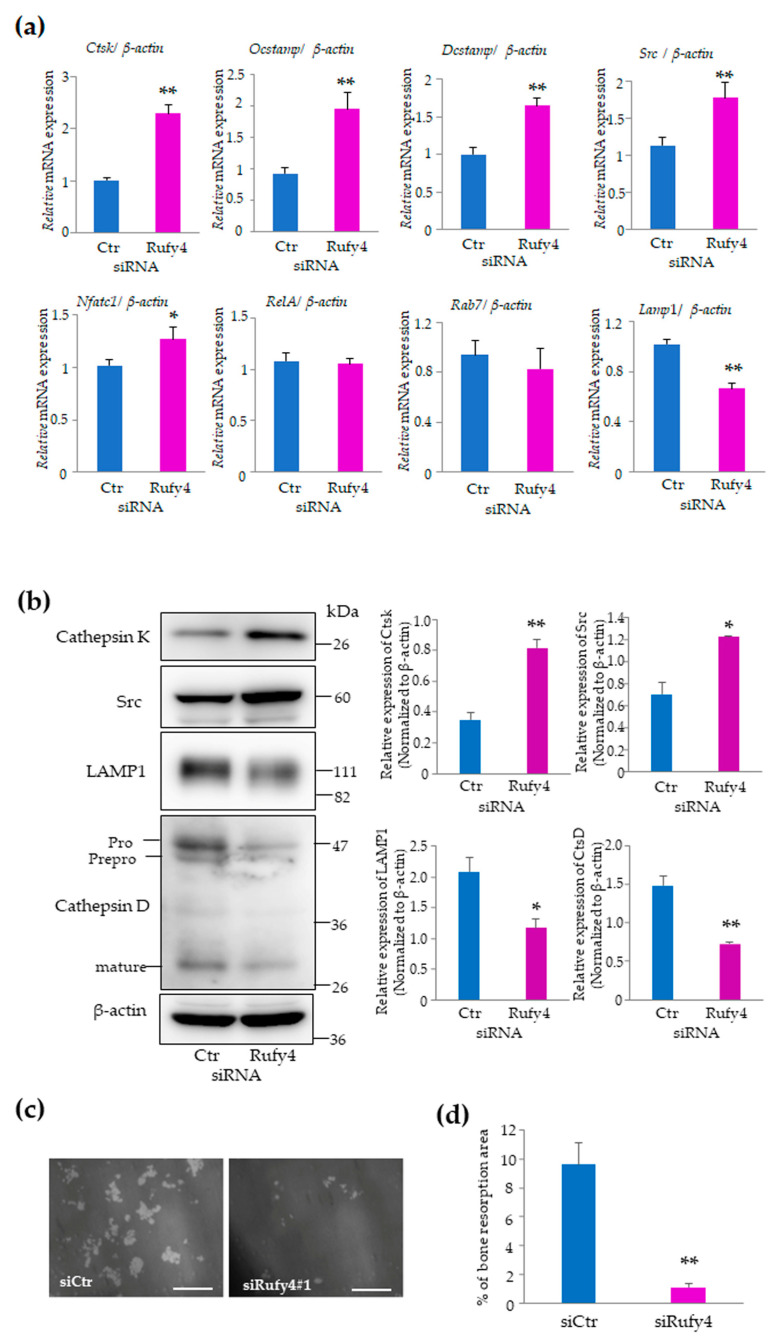
*Rufy4* knockdown promotes osteoclast differentiation but impairs bone resorption. (**a**) *Rufy4* knockdown markedly increases the expression of osteoclast marker genes. Control or *Rufy4*-knockdown RAW-D cells were cultured with 100 ng/mL RANKL for 3 days. After the isolation of mRNA, quantitative real-time PCR was performed. Data are presented as the mean ± SD from 3 independent experiments (* *p* < 0.05, ** *p* < 0.01). (**b**) *Rufy4* knockdown upregulates osteoclast marker proteins. Control or *Rufy4*-knockdown RAW-D cells were cultured with 100 ng/mL RANKL for 3 days and harvested. Lysates were subjected to western blot analysis with specific antibodies against Cathepsin K and c-Src. LAMP1, Cathepsin D, and β-actin (control). Representative immunoblots are shown, and the quantification results are presented as the mean ± SD from 3 independent experiments (* *p* < 0.05, ** *p* < 0.01). (**c**) *Rufy4* knockdown impairs bone resorption. Control and *Rufy4*-knockdown RAW-D cells were seeded on Osteo Assay plates and cultured with 500 ng/mL RANKL for 7–10 days. Images of resorption pits are shown. Scale bar, 200 μm. (**d**) Resorption areas were calculated using the ImageJ software. Data are presented as the mean ± SD from 3 independent experiments (** *p* < 0.01).

To evaluate the differences between control and *Rufy4*-knockdown osteoclasts, we measured the mRNA expression of various osteoclast marker genes using qRT-PCR analysis. The results showed that the expression of *Ctsk*, osteoclast stimulatory transmembrane protein (*Ocstamp*), *Dcstamp*, *Src*, *and Nfatc1* was significantly higher in *Rufy4*-knockdown osteoclasts than in control cells (Figure 2a), suggesting that *Rufy4*-depleted osteoclasts exhibit increased maturation levels compared to control cells. Notably, the expression of *RelA* and *Rab7* remained unchanged, whereas that of lysosomal membrane protein 1 (*Lamp1*) decreased. Similarly, western blot analysis revealed that the protein levels of cathepsin K and Src were markedly increased, while those of LAMP1 and cathepsin D were decreased in *Rufy4*-knockdown osteoclasts compared to control cells (Figure 2b).

To assess the bone resorption activity, we conducted a pit formation assay using control and *Rufy4*-depleted osteoclasts. Surprisingly, osteoclasts lacking *Rufy4* exhibited a significant decrease in resorption activity compared to control osteoclasts (Figure 2c). Furthermore, the bone resorption area generated by *Rufy4*-knockdown osteoclasts was unexpectedly smaller than that generated by control osteoclasts despite the significantly elevated expression of cathepsin K, a cysteine proteinase involved in bone resorption (Figure 2d). These findings suggest that while *Rufy4* depletion enhances osteoclast differentiation, it also disrupts the functional maturation necessary for effective bone resorption.

### 3.3. Rufy4 Deficiency Promotes Large Axial Protrusive Structure Formation

**Figure 3:**
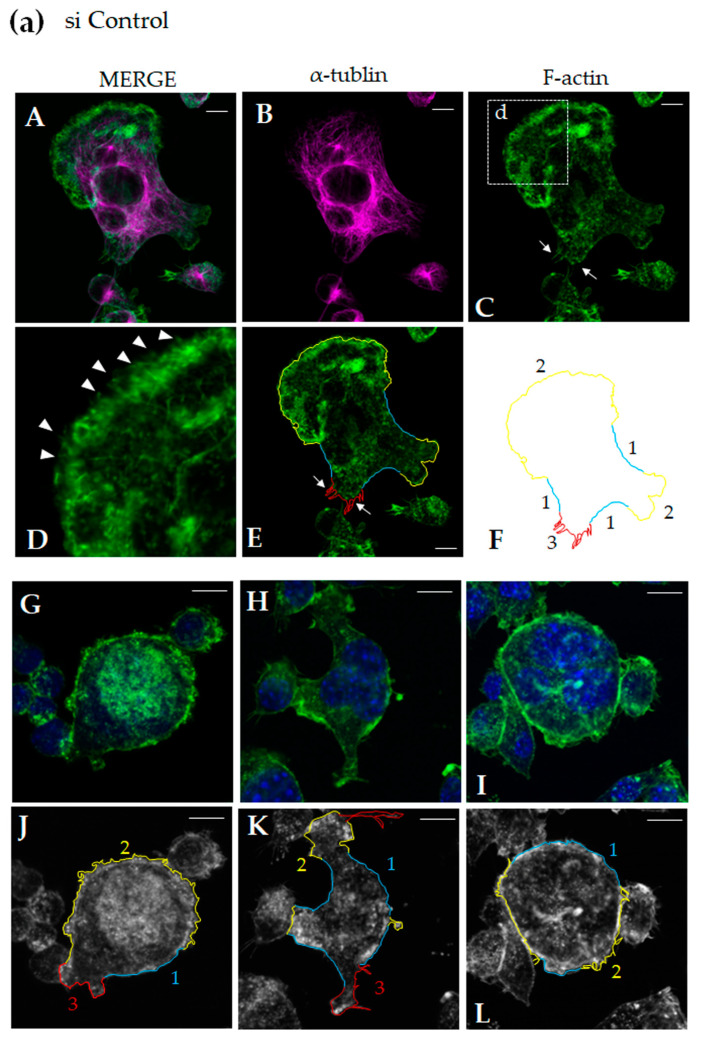

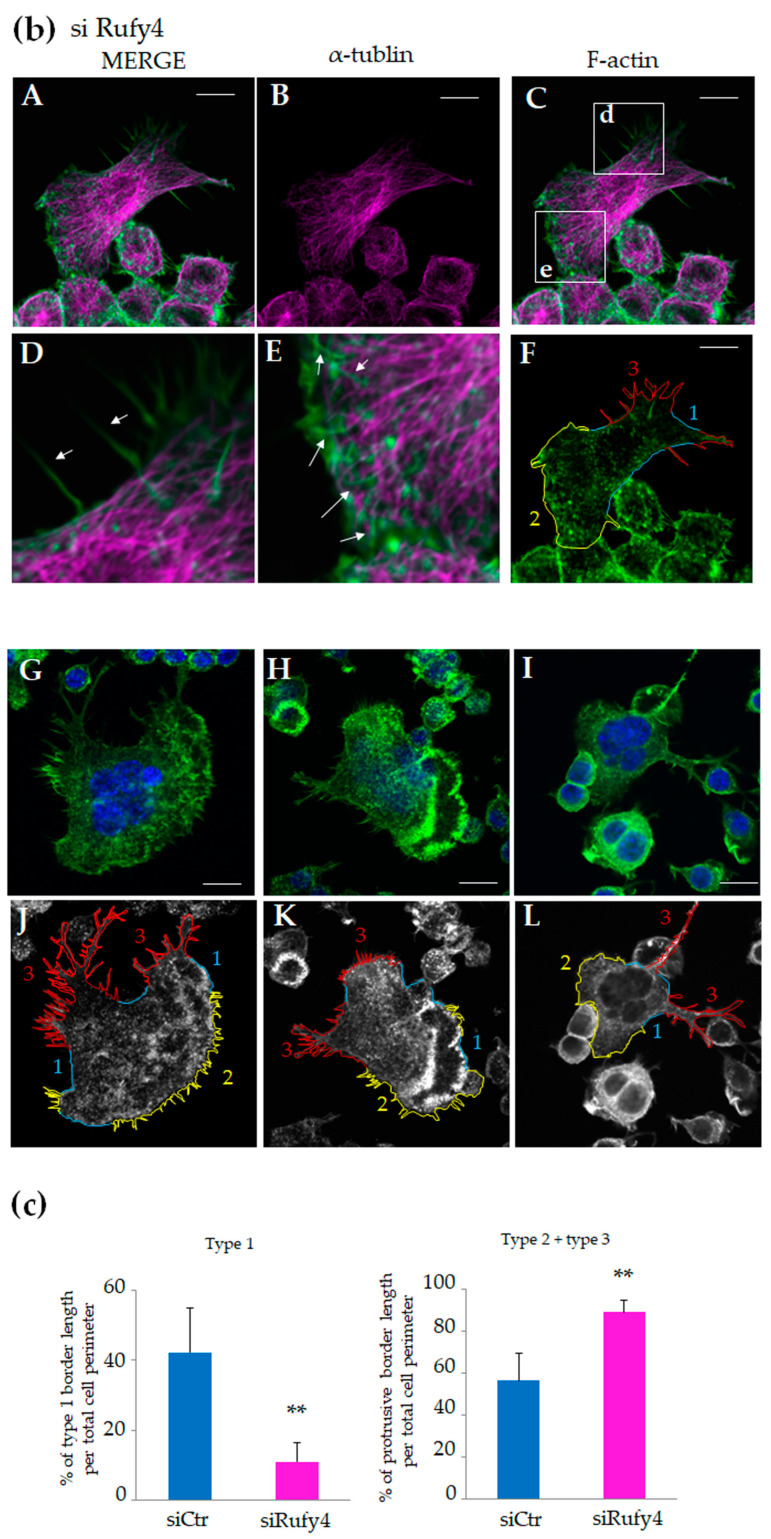
*Rufy4* knockdown promotes large axial protrusive structure formation. Control RAW-D cells or *Rufy4* knockdown RAW-D cells were cultured on glass coverslips with 100 ng/mL RANKL. After 3 days, the cells were fixed and stained with phalloidin (green) and α-tubulin antibody (magenta) and then visualized by confocal microscopy (A–E). (**a**) Control osteoclasts exhibited actin accumulation along the cell periphery (A). Tubulin was distributed throughout the cytoplasm (B). Arrows indicate protrusive structures in the tail-like region (C). High magnification image of the dotted square d in panel C. Arrowheads indicate the presence of hair-like structures (D). The cell contour was traced by blue, yellow, and red lines corresponding to the Type 1, 2, and 3 lines (E). Only the traced lines for (E) are visualized in (F). Representative fluorescence images of control osteoclasts (G–I). Traced lines for (G), (H), and (I) are shown in (J–L). (**b**) *Rufy4*-knockdown osteoclasts exhibited actin distribution along the cell periphery. (A) Tubulin was distributed throughout the cytoplasm (B). High-magnification image of square d and square e in panel C (D and E, respectively). Elongated axial protrusive structures (D, arrows) and actin accumulation (E, arrows) are visualized. The cell contour was traced by blue, yellow, and red lines corresponding to the Type 1, 2, and 3 lines (F). Representative fluorescence images of *Rufy4*-knockdown osteoclasts (G–I). Traced lines for (G–I) are shown in (J–L). Scale bar, 20 μm. (**c**) The relative length of type 1 or type 2 plus 3 curves per whole cell perimeter was compared between the control and *Rufy4*-knockdown osteoclasts. The total number of analyzed cells was 62 and 63 for the control and *Rufy4*-knockdown groups, respectively. All values are presented as mean ± SD (** *p* < 0.01, compared to the control).

According to previous findings, osteoclast-mediated bone resorption depends on the accumulation of actin and the formation of podosomes [38]. To analyze cell shape and actin cytoskeleton, we performed immunofluorescence staining for actin and tubulin in both control and Rufy4-knockdown osteoclasts. In control osteoclasts, accumulation of F-actin was visualized along the cell periphery (Figure 3a, panel A), whereas tubulin was distributed throughout the cytoplasm but not at the cell periphery (Figure 3a, panel B). Several protrusive structures on a tail-like portion (Figure 3a, arrows, panel C) and hair-like structures at the cell periphery were visualized (Figure 3a, arrowheads, panel D). In this study, to compare the cell shape, we traced cell contour and categorized them into three types according to their contour as follows: (Type 1, traced by blue) curves without large protrusive structure, (Type 2, traced by yellow) curves with hair-like protrusive structures, (Type 3, traced by red) protrusive complex curves with a sharp structure more like a needle than hair. The traced lines for (E) were visualized (F). ImageJ measured the length of each curve. Multinucleated osteoclasts (Figure 3a, G,H) and corresponding phalloidin images with traced lines (Figure 3a, J,K) showed fewer type 3 curves in control cells. Conversely, *Rufy4*-knockdown osteoclasts exhibited elongated axial protrusions composed of actin and tubulin (Figure 3b, A–C). Magnification revealed that the elongated axial protrusive structures were primarily composed of actin, with their most apical tips formed by actin polymerization (Figure 3b, D, arrows), supported by α-tubulin just inside the actin tip (Figure 3b, D). Additionally, actin assembly with hair-like structures (Figure 3b, E, arrows) were observed around the periphery of multinucleated osteoclasts. We traced cell contour and measured the length of each curve using ImageJ. Multinucleated osteoclasts (Figure 3b, G,H) and corresponding phalloidin images with traced lines (Figure 3b, J,K) showed abundant type 2 and type 3 curves in *Rufy4*-knockdown cells. The ImageJ analysis of multinucleated osteoclasts revealed a significant decrease in the relative length of the type 1 boundary per whole cell perimeter in *Rufy4*-knockdown cells and, conversely, a significant increase in the total length of type 2 and type 3 boundaries. These findings suggest that impaired bone resorption in *Rufy4*-knockdown osteoclasts may be attributed to abnormal cell morphology caused by abnormal actin polymerization.

### 3.4. Overexpression of Rufy4 Suppresses Osteoclast Differentiation but Enhances Bone Resorption

**Figure 4:**
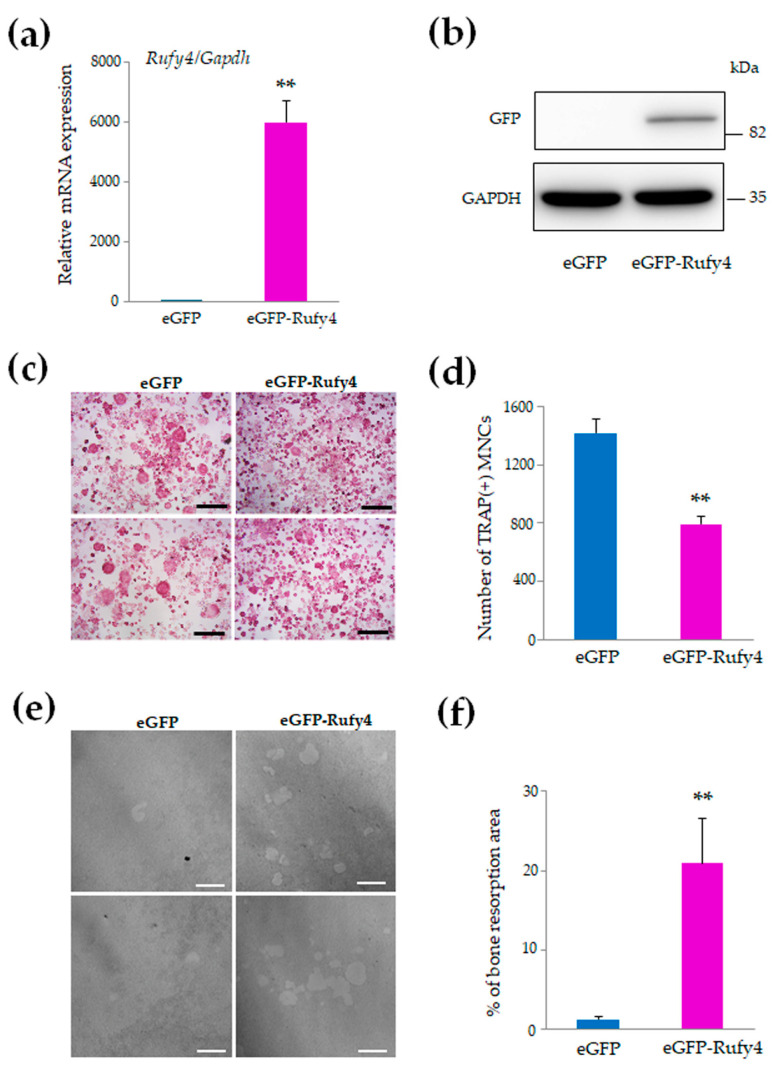

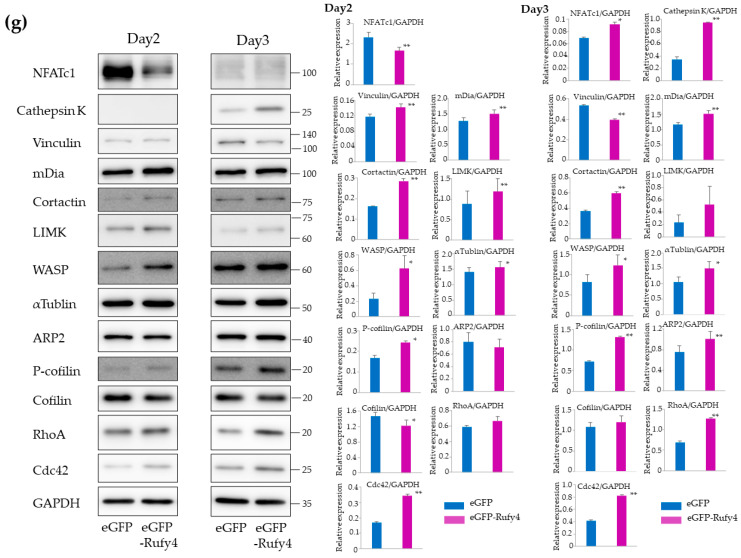
Overexpression of eGFP-*Rufy4* inhibits osteoclast formation and stimulates bone resorption. (**a**) Quantitative RT-PCR analysis of *Rufy4* mRNA expression in RAW-D cells expressing either eGFP or eGFP-*Rufy4*. Data are presented as the mean ± SD from 3 independent experiments (** *p* < 0.01). (**b**) Western blot analysis of RAW-D cells expressing eGFP or eGFP-*Rufy4*. Cell lysates were subjected to western blot analysis using anti-GFP or anti-GAPDH antibodies. (**c**) TRAP staining of eGFP- and eGFP-*Rufy4*-overexpressing osteoclasts. eGFP- or eGFP-*Rufy4*-overexpressing RAW-D cells were stimulated with 100 ng/mL RANKL for 3 days and then fixed and stained for TRAP. Scale bar, 50 μm. (**d**) Number of TRAP-positive multinucleated cells. Data are presented as the mean ± SD from 3 independent experiments (** *p* < 0.01). (**e**) Bone resorption area of eGFP or eGFP-*Rufy4*-overexpressing osteoclasts. Cells were seeded onto Osteo Assay Stripwell Plates containing 500 ng/mL RANKL. The images show the bone resorption area for each osteoclast. Scale bar, 20 μm. (**f**) The bone resorption area was determined using the ImageJ software. Data are presented as the mean ± SD from 3 independent experiments (** *p* < 0.01). (**g**) Western blot analysis of eGFP or eGFP-*Rufy4*-overexpressing RAW-D cells cultured with 100 ng/mL RANKL for 2 or 3 days. Cells were harvested on the indicated day, and lysates were subjected to western blot analysis with specific antibodies against various proteins involved in osteoclast differentiation and actin polymerization, with GAPDH as a control. Representative immunoblots are shown, and the quantification results are presented as the mean ± SD from 3 independent experiments (* *p* < 0.05, ** *p* < 0.01).

To explore the physiological functions of *Rufy4* during osteoclast differentiation, we conducted overexpression experiments using a vector encoding enhanced green fluorescent protein (eGFP)-*Rufy4* or eGFP alone (control, empty vector) in RAW-D cells. The mRNA expression and protein levels of *Rufy4* were determined by qRT-PCR and western blot analysis, respectively. qRT-PCR results showed that *Rufy4* mRNA expression in eGFP-*Rufy4*-overexpressing RAW-D cells was approximately 6000-fold higher than that in the control-transfected cells (Figure 4a). Furthermore, western blot analysis using an anti-GFP antibody detected the eGFP-Rufy4 protein as a major band with a molecular mass of approximately 92 kDa (Figure 4b). Next, we investigated the effects of *Rufy4* on RANKL-induced osteoclastogenesis (100 ng/mL) by TRAP staining.

The results showed that *Rufy4* overexpression abolished osteoclast differentiation after stimulation with RANKL for 3 days (Figure 4c). Although both control cells and eGFP-*Rufy4*-overexpressing cells were TRAP-positive, the number of multinucleated osteoclasts was significantly reduced in the latter group (Figure 4d).

To further evaluate bone resorption in the control and *Rufy4*-overexpressing osteoclasts, we performed a pit formation assay and observed a significant increase in the bone resorption area in *Rufy4*-overexpressing osteoclasts compared to control osteoclasts (Figure 4e,f). These findings suggest that *Rufy4* overexpression in RAW-D cells inhibits osteoclast differentiation while simultaneously increasing bone resorption activity. This dual effect highlights *Rufy4*’s complex role in regulating both osteoclast formation and function.

### 3.5. Rufy4 Overexpression Upregulates Cathepsin K and Proteins Involved in Actin Polymerization

We examined the expression of osteoclast markers and various proteins involved in actin polymerization and bone resorption using western blot analysis. The results revealed a decrease in the levels of NFATc1, vinculin, and cofilin, while several other proteins, including cathepsin K, mDia, cortactin, LIMK, WASP, αTublin, ARP2, P-cofilin, RhoA, and cdc42, were upregulated in Rufy4-overexpressing osteoclasts (Figure 4g). Thus, *Rufy4* overexpression leads to the upregulation of cathepsin K and several proteins involved in actin polymerization, further supporting the enhanced bone resorption activity observed in *Rufy4*-overexpressing osteoclasts.

### 3.6. Overexpression of a RUN-Domain Deletion Mutant of Rufy4 Promotes Osteoclast Formation but Suppresses Bone Resorption

**Figure 5:**
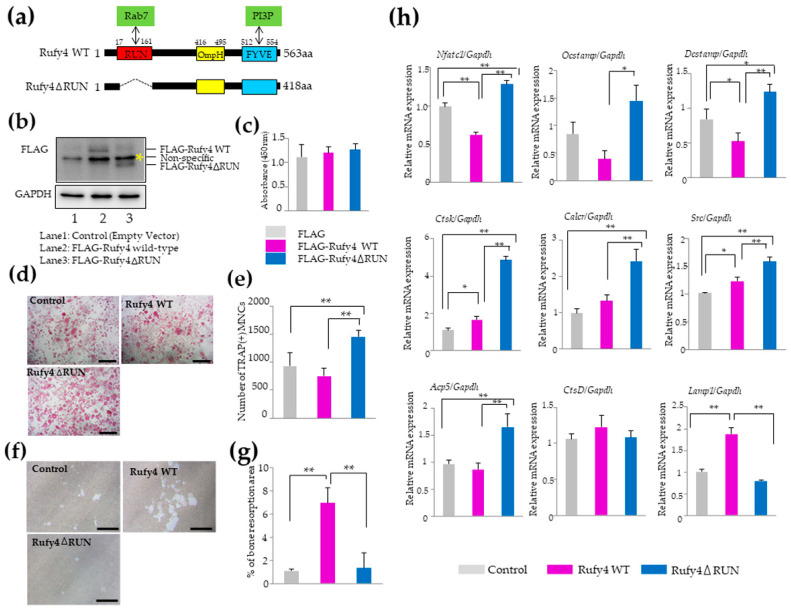

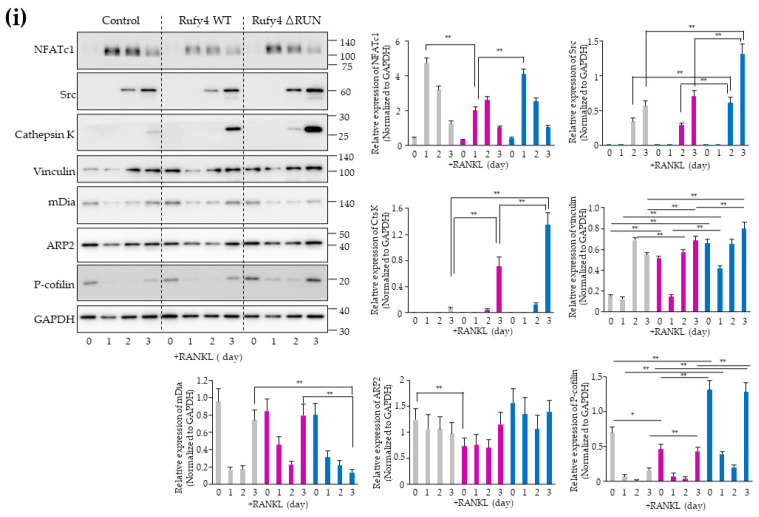
A RUN-domain deletion mutant of *Rufy4* stimulates osteoclast formation but impairs bone resorption. (**a**) Schematic representation of *Rufy4* wild-type and RUN-domain deletion mutants. RUN, OmpH, and FYVE domains are shown in red, yellow, and blue, respectively. Recombinant Rufy4 was expressed as an N-terminal 1 × FLAG-tagged fusion protein. (**b**) Western blot analysis of RAW-D cells expressing the control (empty vector), *Rufy4* wild-type, and RUN-domain deletion mutants. Yellow asterisk indicates non-specific binding of antibody (**c**) Cell viability was assessed using the Cell Counting Kit-8. Data are presented as mean ± SD from 3 independent experiments. (**d**) TRAP staining of osteoclasts overexpressing the control, *Rufy4* wild-type, and RUN-domain deletion mutants. Control, *Rufy4* wild-type, and RUN-domain deletion mutant-overexpressing RAW-D cells were stimulated with 100 ng/mL RANKL for 3 days. The cells were then fixed and stained for TRAP. Scale bar, 100 μm. (**e**) Number of TRAP-positive multinucleated cells. Data are presented as mean ± SD from three independent experiments (** *p* < 0.01). (**f**) Bone resorption areas of osteoclasts overexpressing control, *Rufy4* wild-type, and RUN-domain deletion mutant, seeded onto Osteo Assay Stripwell Plates containing 500 ng/mL RANKL. The images show the bone resorption area for each osteoclast. Scale bar, 100 μm. (**g**) The bone resorption area was determined using the ImageJ software. Data are presented as mean ± SD from three independent experiments (** *p* < 0.01). (**h**) The RUN-domain deletion mutant overexpressing osteoclasts exhibited a significantly increased expression of osteoclast marker genes. RAW-D cells overexpressing the control or *Rufy4* wild-type and RUN-domain deletion mutant were cultured with 100 ng/mL RANKL for 3 days. Following mRNA isolation, quantitative real-time PCR was performed. Data are presented as mean ± SD from three independent experiments (* *p* < 0.05, ** *p* < 0.01). (**i**) Western blot analysis of control (empty vector)-, *Rufy4* wild-type-, and RUN-domain deletion mutant-overexpressing RAW-D cells cultured with 100 ng/mL RANKL for 3 days. Cells were harvested on the indicated day, and lysates were subjected to western blot analysis with specific antibodies against various proteins involved in osteoclast differentiation and actin polymerization, with GAPDH as a control. Representative immunoblots are shown, and the quantification results are presented as mean ± SD from three independent experiments (* *p* < 0.05, ** *p* < 0.01).

Bone resorption by osteoclasts depends on the secretion of cathepsin K [12], which occurs through the ruffled border, a specific region regulated by Rab7 [13]. The Rufy4 protein is composed of the RUN, OmpH, and FYVE domains (Figure 5a). Since Rab7 is known to interact with the RUN domain [6], we generated a FLAG-tagged mutant lacking the RUN domain (Figure 5b) to elucidate the mechanism by which *Rufy4* regulates osteoclastogenesis and bone resorption. There was no significant difference in cell viability (Figure 5c). Compared to control cells or those overexpressing FLAG-tagged wild-type *Rufy4*, TRAP staining results revealed that the *Rufy4*-RUN domain deletion mutant promoted osteoclast differentiation (Figure 5d).

The number of TRAP-positive multinucleated cells was significantly increased in cells overexpressing the *Rufy4*-RUN domain deletion mutant (Figure 5e). According to the Student’s *t*-test, cells overexpressing FLAG-tagged wild-type RUFY4 showed a significant reduction in the number of TRAP-positive osteoclasts compared to control cells (Supplementary Figure S1). Consistent with the results obtained for GFP-*Rufy4*-overexpressing osteoclasts, FLAG-tagged wild-type *Rufy4*-overexpressing osteoclasts exhibited accelerated bone resorption. However, the RUN domain-deletion mutant failed to display the enhanced bone resorption typically observed in wild-type *Rufy4*-overexpressing osteoclasts (Figure 5f,g). To examine the function of the RUN domain in Rufy4, we compared osteoclast marker gene expression among control, wild-type *Rufy4*, and *Rufy4* RUN domain-deletion mutant-overexpressing osteoclasts. In wild-type *Rufy4*-expressing osteoclasts, the mRNA expression of *Nfatc1*, *Ocstamp*, and *Dcstamp* decreased, while that of *Ctsk*, *Calcr*, and *Src* increased, compared to control cells. Conversely, the expression of *Nfatc1*, *Ocstamp*, *Dcstamp*, *Ctsk*, *Calcr*, and *Src* was notably augmented in osteoclasts expressing the RUN domain-deficient mutant. Moreover, *Acp5* (TRAP) expression was significantly amplified in cells expressing the RUN domain-deficient mutant, although no discernible difference was observed in *CtsD* expression. Additionally, *Lamp1* expression was significantly elevated only in cells expressing wild-type *Rufy4* (Figure 5h). Despite the suppression of bone resorption by osteoclasts overexpressing the RUN domain-deficient mutant, there was a significant increase in the expression of cathepsin K, a protein essential for bone resorption, at the mRNA level. To confirm this inconsistency, western blot analysis was conducted to compare the levels of several osteoclast differentiation marker proteins, such as NFATc1 and Src, as well as cathepsin K, and several proteins involved in podosome formation.

According to the qRT-PCR analysis, the highest protein expression of cathepsin K, Src, and phosphorylated-cofilin was observed in osteoclasts that overexpressed the RUN domain-deficient mutant on day 3 following RANKL stimulation (Figure 5i). These findings suggest that the RUN-domain deletion mutant of *Rufy4* promotes osteoclast formation but fails to enhance bone resorption, unlike wild-type *Rufy4*, highlighting the critical role of the RUN domain in osteoclast functional maturation and bone resorption.

### 3.7. Overexpression of Wild-Type Rufy4 Induces Podosome Formation, Whereas That of the RUN-Domain Deletion Mutant Impedes Podosome Formation and Promotes Protrusive Structures

**Figure 6:**
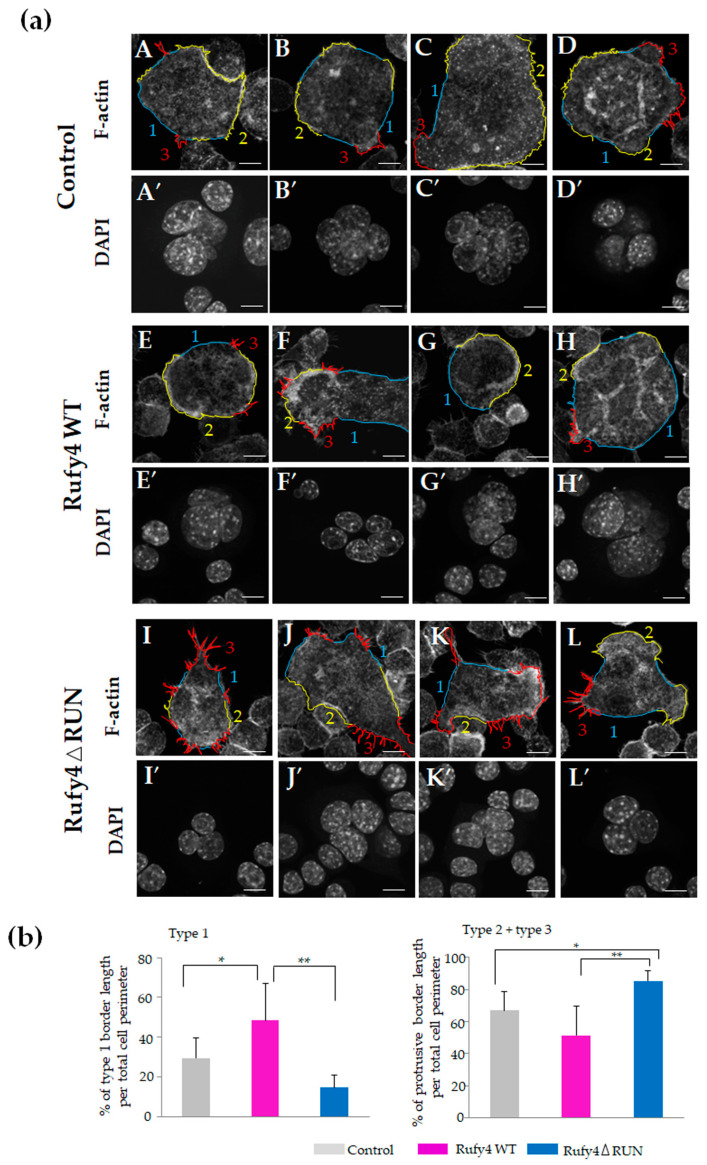

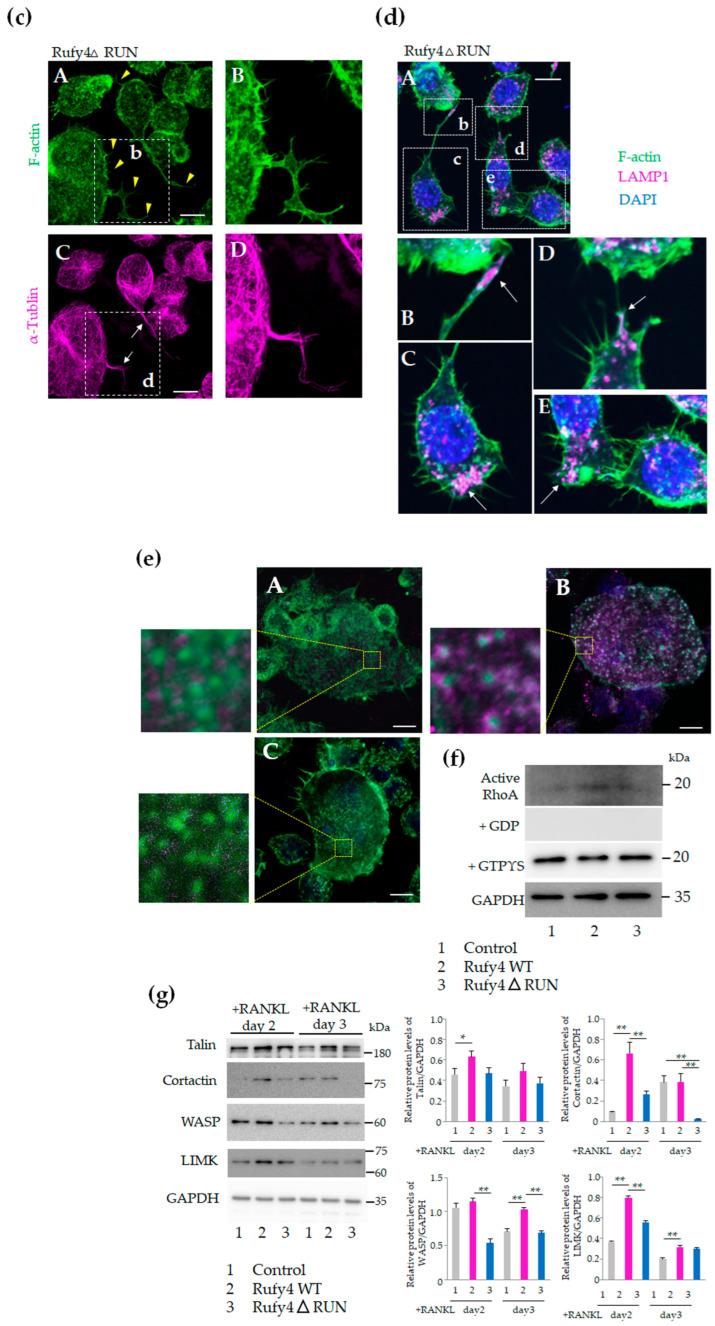
*Rufy4* wild-type-overexpressing osteoclasts exhibit extensive F-actin assembly, whereas RUN-domain-deficient mutant-expressing osteoclasts show enhanced formation of protrusive structures. (**a**) RAW-D cells overexpressing the control (A–D, A′–D′), FLAG-tagged *Rufy4* wild-type (E–H, E′–H′), and FLAG-tagged *Rufy4* RUN-domain deletion mutant (I–L, I′–L′) were cultured on glass coverslips with 100 ng/mL RANKL. After 3 days, the cells were fixed, stained with phalloidin and DAPI, and visualized using confocal microscopy. Control multinucleated osteoclasts exhibit F-actin assembly (A–D). *Rufy4* wild-type-overexpressing osteoclasts showed abundant F-actin assemblies, such as belts (E–H), whereas *Rufy4* RUN-domain deletion mutant-overexpressing osteoclasts showed elongated axial protrusive structures (I–L). Cell contour was traced by blue for Type 1 lines, yellow for Type 2 lines, and red for Type 3 lines, using phalloidin images. Scale bar, 20 μm. (**b**) The relative length of type 1 or type 2 plus 3 curves per whole cell perimeter was compared. The total number of analyzed cells was 54, 66, and 68 for control and *Rufy4* wild-type-overexpressing osteoclasts and *Rufy4* RUN-domain deletion mutant-overexpressing osteoclasts, respectively. All values are presented as the mean ± SD (* *p* < 0.05, ** *p* < 0.01). (**c**) RAW-D cells overexpressing the *Rufy4* RUN domain deletion mutant were cultured on glass coverslips with 100 ng/mL RANKL. After 3 days, the cells were fixed and stained with phalloidin (green) and α-tubulin antibody (magenta) and then visualized by confocal microscopy. Polymerization of F-actin (A, yellow arrowheads) and tubulin (C, white arrows) is observed. (B) Higher magnification image of the dotted square b in A. (D) Higher magnification image of the dotted square d in A. Scale bar, 20 μm. (**d**) RAW-D cells overexpressing the *Rufy4* RUN domain deletion mutant were cultured on glass coverslips with 100 ng/mL RANKL. After 3 days, the cells were fixed and stained with phalloidin (green), LAMP1 (magenta), and DAPI (blue) and visualized using confocal microscopy (A) (B–E) Higher magnification images of the dotted square (b–e) in (A), respectively. LAMP1 was observed at the cell periphery and tips of the moving tail and bridge (arrows). Scale bar, 20 μm. (**e**) RAW-D cells overexpressing the control (A), FLAG-tagged *Rufy4* wild-type (B), and FLAG-tagged *Rufy4* RUN-domain deletion mutant (C) were cultured on glass coverslips with 100 ng/mL RANKL. After 3 days, the cells were fixed, stained with phalloidin (green), anti-vinculin antibody (magenta), and DAPI (blue), and visualized using confocal microscopy. High-magnification images of the dotted squares are shown on the left side of each image. Scale bar, 20 μm. (**f**) Blot analyses of GTP-bound RhoA in osteoclasts overexpressing control, FLAG-tagged *Rufy4* wild-type, and FLAG-tagged *Rufy4* RUN-domain deletion mutant. The cells were cultured with 100 ng/mL RANKL. After 3 days, cell extracts with the same amounts of protein were subjected to Rho GTPase pull-down experiments. GDP and GTPγS were used as the negative and positive controls, respectively. (**g**) Western blot analysis of control-, *Rufy4* wild-type-, and RUN-domain deletion mutant-overexpressing RAW-D cells cultured with 100 ng/mL RANKL for 2 or 3 days. Cells were harvested, and lysates were subjected to western blot analysis with specific antibodies against various proteins involved in actin polymerization and podosome formation, with GAPDH as a control. Representative immunoblots are shown, and the quantification results are presented as mean ± SD from three independent experiments (* *p* < 0.05, ** *p* < 0.01).

To investigate why Rufy4 wild-type osteoclasts exhibited accelerated bone resorption and RUN domain-deficient mutant-overexpressing osteoclasts showed impaired bone resorption, we compared actin distribution and cell morphology using immunofluorescence staining for actin and tubulin. To compare the cell shape, using phalloidin images, we traced cellular contour and measured the length of type 1, type 2, and type 3 curves using ImageJ software (Figure 6a,b). In control, multinucleated osteoclasts, actin assembles like clusters were detected (Figure 6a, A–D for F-actin and A′–D′ for the corresponding nuclea images). Wild-type Rufy4-overexpressing osteoclasts exhibited more prominent actin polymerization with broad belt-like structures than control cells (Figure 6a, E–H for F-actin and E′–H′ for the corresponding nuclei), whereas RUN domain-deficient mutant-overexpressing osteoclasts displayed reduced actin assembly and increased protrusive structures, classified as type 3 curves (Figure 6a, I–L for F-actin and I′–L′ for the corresponding nuclea images). ImageJ analysis of multinucleated osteoclasts revealed a significant increase in the relative length of the type 1 boundary per whole cell perimeter in wild-type Rufy4-overexpressing osteoclasts compared to that of control osteoclasts or that of RUN domain-deficient mutant-overexpressing osteoclasts (Figure 6b, left). Conversely, a significant increase in the relative length of type 2 plus 3 boundaries per whole cell perimeter was observed in RUN domain-deficient mutant-overexpressing osteoclasts compared to that of control or that of wild-type Rufy4-overexpressing osteoclasts (Figure 6b, right).

Three days after the addition of RANKL, the RUN domain-deficient mutant-overexpressing osteoclasts displayed notable long protrusions and moving tail formation, especially in mononuclear cells (Figure 6c). Actin polymerization was observed at the tips of these protrusions and tails (Figure 6c, A, yellow arrowheads), with tubulin extending slightly inward (Figure 6c, C, arrows). Interestingly, LAMP1 was detected at the tips of the moving tail (Figure 6d, B arrow), the bridge connecting cell to cell (Figure 6d, D arrow), and the opposite side where the moving tail or bridge was extending (Figure 6d, C, and E arrows). Given that actin cytoskeleton reorganization is regulated by podosome formation and the activation of Rho small GTPases, immunofluorescence staining was performed to observe the podosome structures consisting of actin-core surrounded by a ring of vinculin, and RANKL-induced RhoA activation was assessed. In control osteoclasts, some actin cores were surrounded by vinculin (Figure 6e, A); however, in Rufy4 wild-type-overexpressing osteoclasts, most actin-cores were surrounded by ring-like structures of vinculin, suggesting that podosome formation is enhanced by Rufy4 overexpression (Figure 6e, B). In contrast, in RUN domain-deficient mutant-overexpressing osteoclasts, the actin-cores were not surrounded by vinculin (Figure 6e, C). Moreover, western blotting results showed relatively increased levels of GTP-bound RhoA in Rufy4 overexpressing osteoclasts (Figure 6f), suggesting that Rufy4 promotes reorganization of the actin cytoskeleton. Consistent with the increased level of GTP-bound RhoA, the highest protein levels of Talin, Cortactin, WASP, and LIMK, which are involved in podosome formation, were observed in Rufy4 overexpressing osteoclasts (Figure 6g).

Collectively, these findings suggest that overexpression of wild-type Rufy4 promotes podosome formation, which is crucial for bone resorption, while overexpression of the RUN-domain deletion mutant disrupts this process and increases abnormal protrusive structure development, underscoring the crucial role of the RUN domain in maintaining osteoclast morphology and functionality.

### 3.8. RUN Domain Deficiency Promotes Development of Protrusive Structures During Osteoclastogenesis

**Figure 7:**
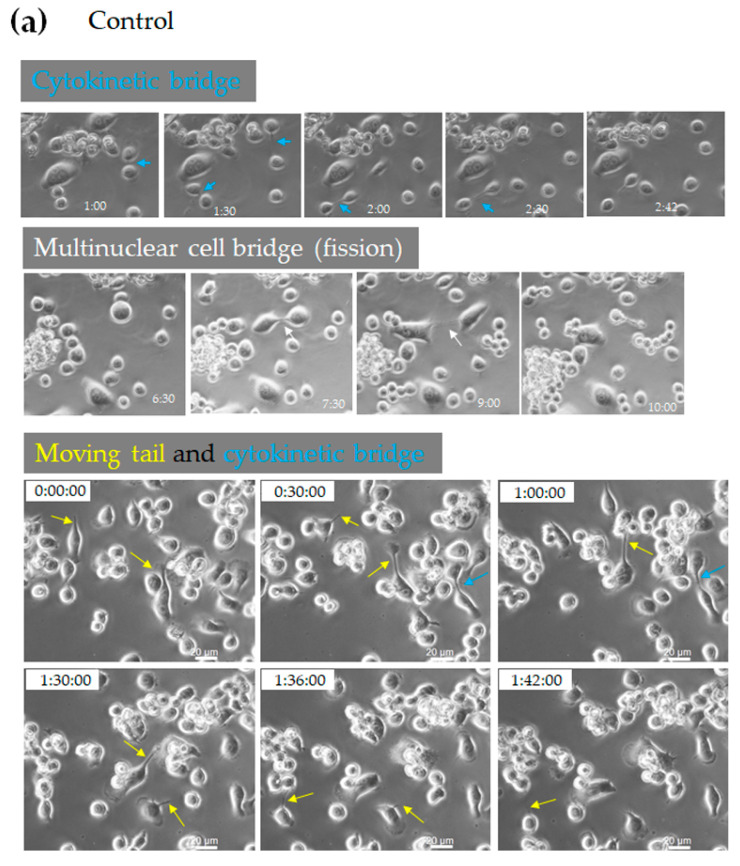

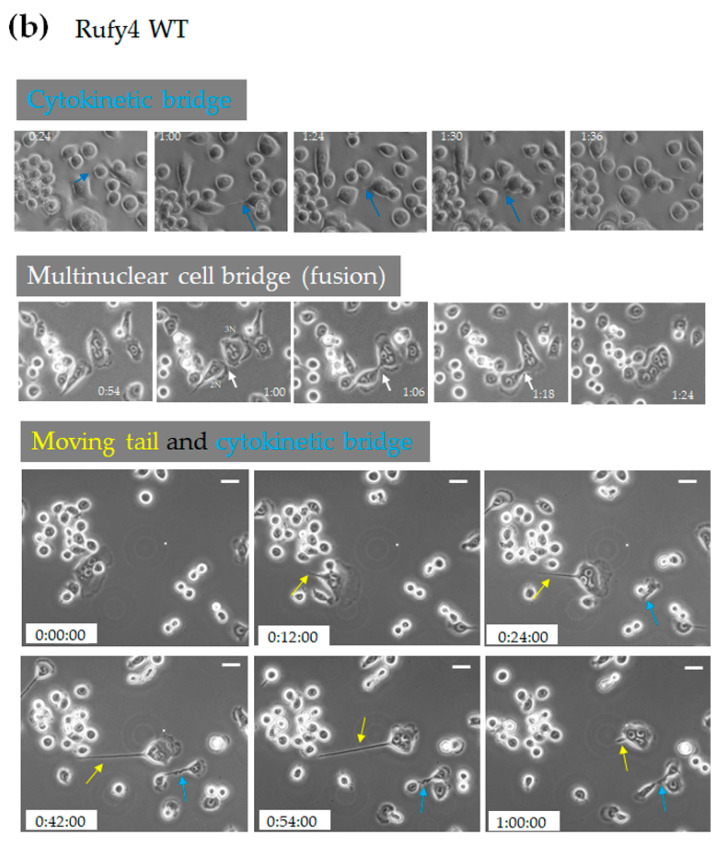

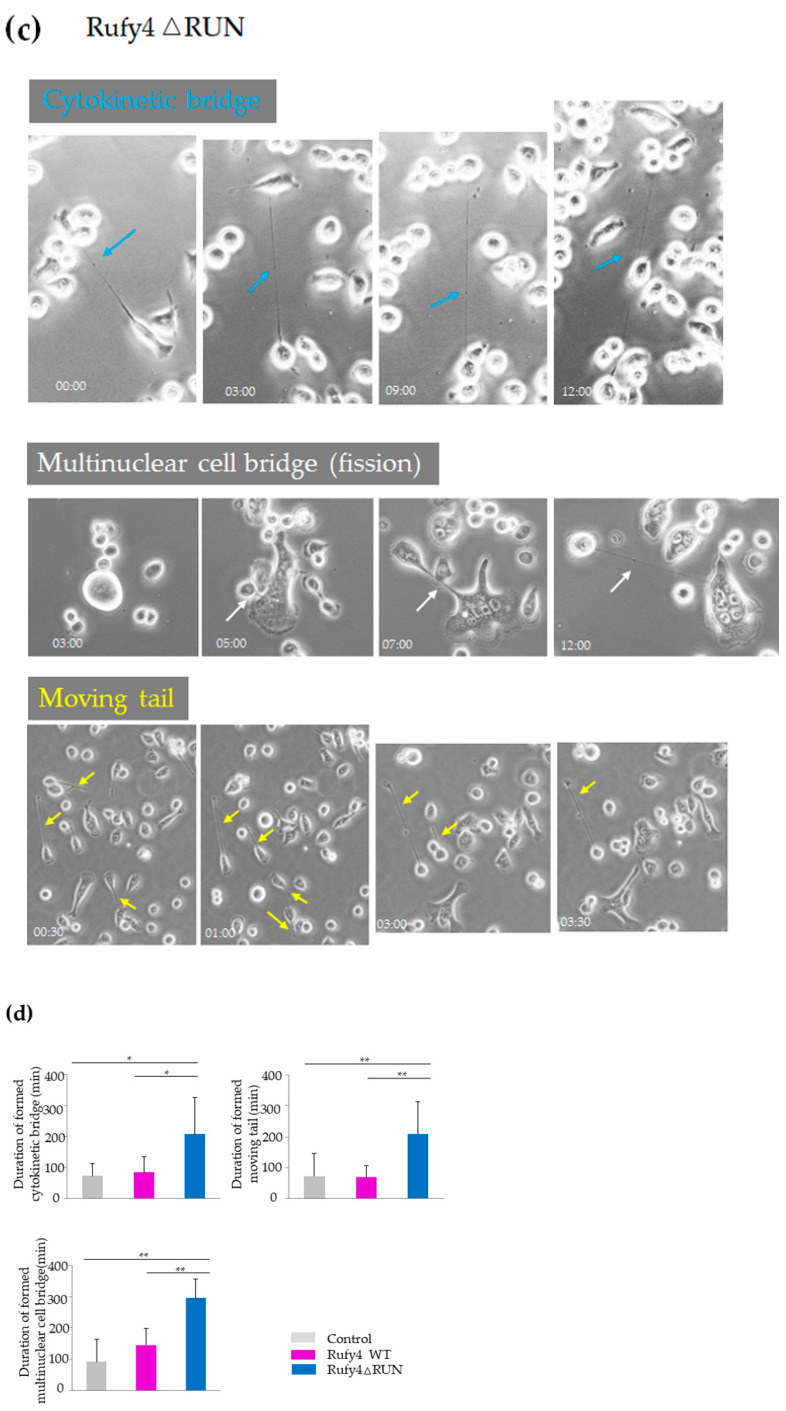
A RUN-domain deletion mutant of *Rufy4* exhibits long-lasting bridge and tail formation. RAW-D cells expressing the control (empty vector, (**a**)), *Rufy4* wild-type (**b**), and RUN-domain deletion mutant (**c**) were cultured on 35 mm plastic culture dishes with 100 ng/mL RANKL for 48 h, and live-cell phase contrast imaging was performed every 6 min for 12 h. Time-lapse images showing bridges and tails (arrows) are representative of five independent experiments. (**d**) The formation and duration of bridges and tails were quantified using live-cell video data. The total numbers of analyzed cells for the duration time of the cytokinetic bridge were 35, 30, and 65; for the duration time of moving tail, they were 63, 45, and 66; and for duration time of multinuclear cell bridge were 35, 45, and 35 for control and *Rufy4* wild-type-overexpressing osteoclasts, and *Rufy4* RUN-domain deletion mutant-overexpressing osteoclasts, respectively. Data are presented as mean ± SD (* *p* < 0.05, ** *p* < 0.01).

To analyze the chronological changes of the protrusive structures of control, wild-type *Rufy4*-, and RUN domain-deficient mutant-expressing osteoclasts, time-lapse phase-contrast imaging was performed using a Keyence microscope (BZ-X800, Keyence, Osaka, Japan), following a two-day stimulation with RANKL. Time-lapse imaging recorded at 6-min intervals for 12 h showed that the formation of extended protrusive structures, including moving tails and cytokinesis (bridges between mononuclear cells), was sustained for longer periods in osteoclasts overexpressing the RUN domain-deficient mutant compared to control cells or wild-type *Rufy4*-overexpressing cells (Figure 7a–d). Moreover, cell bridges between multinucleated cells and mononuclear cells (which then proceed to fission) and cell bridges between multinuclear cells and mononuclear cells or multinuclear cells (which then undergo fusion) were sustained for longer periods. Time-lapse videos of these cells are provided (Supplementary Videos 1–3). These findings suggest that the RUN domain of Rufy4 plays a crucial role in osteoclast morphogenesis.

## 4. Discussion

This study showed dynamic changes in *Rufy4* expression during osteoclast differentiation, with knockdown experiments revealing its significant role in promoting osteoclast differentiation and upregulating key osteoclast marker genes such as *Ctsk*, *Ocstamp*, *Dcstamp*, and *Src*. However, this elevation in osteoclast activity paradoxically led to impaired bone resorption despite increased cathepsin K levels. In contrast, wild-type *Rufy4* expression downregulated osteoclast marker genes, such as *Nfatc1* and *Dcstamp*, and hindered osteoclast formation, suggesting a complex regulatory role. Interestingly, both eGFP-tagged and FLAG-tagged wild-type *Rufy4* induced increased cathepsin K expression and robust bone resorption activity, underscoring its pivotal role in osteoclast function. From a structural perspective, the Rufy4 protein possesses a crucial RUN domain that is thought to interact with Rab7. Notably, the expression of a mutant lacking this domain resulted in a significant reduction in osteoclast-mediated bone resorption despite elevated gene and protein levels of cathepsin K. These findings unequivocally highlight the indispensable role of Rufy4’s RUN domain in facilitating bone resorption.

These findings suggest that the reduced expression of LAMP1 in *Rufy4*-knockdown osteoclasts might impede endosome/lysosome maturation, affecting the development of ruffled borders. Immunofluorescence imaging further revealed diminished actin assembles and enhanced formation of protrusive structures in *Rufy4*-knockdown osteoclasts compared to controls. The unexpected outcome of impaired bone resorption, despite elevated cathepsin K protein levels, may imply a disruption in normal vesicle transport and ruffled border formation. In contrast, osteoclasts overexpressing wild-type *Rufy4* exhibited increased bone resorption compared to control osteoclasts, potentially attributed to enhanced actin assembles to promote podosome formation and cathepsin K secretion. This inference is supported by the increased levels of molecules involved in actin polymerization, such as mDia, Cortactin, WASP, and p-cofilin, as well as RhoA and cdc42, pivotal in cell adhesion and sealing zone formation. Furthermore, immunofluorescence imaging revealed a decrease in actin assembles in *Rufy4*-knockdown osteoclasts compared to controls. Instead, *Rufy4*-knockdown osteoclasts exhibited elongated protrusive structures with F-actin localized at the tip along the tubulin axis.

During the preparation of this paper, a study by Kim et al. on *Rufy4*-knockout mice was published [39]. Their findings were consistent with ours, suggesting *Rufy4*’s role in promoting bone resorption and highlighting the significance of the RUN domain in this process. They observed reduced secretion of cathepsin K in *Rufy4*-knockout osteoclasts, indicating *Rufy4*’s involvement in regulating bone resorption through its RUN, FYVE, and CC domains. In our study, the siRNA-mediated suppression of *Rufy4* expression during osteoclast differentiation led to a slight increase in the expression of NFATc1, the master regulator of osteoclasts, despite no difference in NF-κB expression. In contrast, both eGFP-tagged and FLAG-tagged *Rufy4*-overexpressing osteoclasts exhibited reduced NFATc1 expression. However, Kim et al. reported that *Rufy4* did not affect osteoclast differentiation, while our findings suggest that *Rufy4* affects osteoclast differentiation via its RUN domain. The discrepancy in the outcomes for osteoclast differentiation may be attributed to the fact that Kim et al. utilized bone marrow macrophages (BMM) derived from *Rufy4*-knockout mice, whereas our study involved siRNA transfection into RAW-D cells, resulting in a 60% reduction in expression. Furthermore, in the overexpression experiments, Kim et al. selected retrovirus-infected BMM cells with puromycin for only 3 days, whereas we selected retrovirus-infected RAW-D cells with puromycin for a longer duration of 2 weeks.

Overexpression of *Rufy4* RUN domain-deficient mutant resulted in high levels of phosphorylated-cofilin, indicating enhanced reorganization of the actin cytoskeleton [40]. Time-lapse live cell imaging revealed osteoclasts overexpressing RUN-domain-deficient *Rufy4* adhered to neighboring cells via protrusions for extended periods. Moreover, cytokinesis, a final stage of the cell division cycle, was observed, but it took a long time for cytoplasmic division to be completed.

This suggests that the dissociation and promotion of actin and tubulin polymerization may have been impeded. Phosphorylated-cofilin likely contributes to maintaining abundant elongated filopodia around the cell and relatively thick protrusions with tubulin in the center. Contact via protrusive structures with surrounding cells may promote cell-cell interconnections and increase opportunities for fusion, potentially leading to enhanced osteoclastogenesis.

Fujiwara et al. previously reported that PLEKHM1 interacts with Rab7 in osteoclasts and that microtubule-mediated lysosomal positioning is crucial for ruffled border formation and bone resorption [41]. Given that Rufy4 binds to Rab7 [6] and PLEKHM1, based on previous studies using mass spectrometry [7], it is plausible that Rufy4 participates in tubulin-mediated lysosomal positioning. Recently, a study using HeLa cells reported that Rufy4, in cooperation with Rufy3, functions as an effector of Arl8, a small GTPase, and promotes coupling of endolysosomes to dynein-dynactin for their retrograde trafficking [8]. In the current study, immunofluorescence staining showed that LAMP1 was localized at the tips of protrusions in osteoclasts overexpressing RUN domain-deficient Rufy4. Wild-type Rufy4 colocalizes with LAMP2. However, RUN-domain-deficient Rufy4 doesnot (Supplementary Figure S2). These results suggest that RUN-domain-deficient Rufy4 is unable to bind to Rab7 for retrograde endolysosomal transport, instead transporting LAMP1 to the tip of the protrusion, the plus end of tubulin. This abnormality in the intracellular transportation process affects the formation of ruffled borders and suppresses the secretion of cathepsin K outside the cell, as reported by Kim et al., potentially resulting in the inhibition of bone resorption. Rufy4, lacking the RUN domain, loses its ability to interact with Rab7 and other target molecules via this domain, potentially increasing interactions via the FYVE domain.

The round structures in the bridge observed in time-lapse imaging appear to transport cargo between cells. At the late stage of cytokinesis, the midbody marker protein CRIK was detected by confocal microscopy [42] (Supplementary Figure S3), although midbody-like structures are also observed in bridges formed between multinucleated osteoclasts and mononucleated cells. The current study did not identify the specific components or molecules transported via cellular protrusions; it is possible that the protrusions serve as a means of delivering membrane components and molecules involved in their formation.

Tuberous sclerosis complex 1 (TSC1) regulates podosome organization and bone resorption via mTORC1 and Rac1/Cdc42 in osteoclast [43]. Although the activation of RhoA was detected in wild-type Rufy4 overexpressing osteoclasts in this study, autophagy regulator Rufy4 might affect the TSC1 pathway.

In summary, our findings unveil *Rufy4* as a previously unidentified regulator of osteoclast differentiation and function. *Rufy4* knockdown enhances osteoclast differentiation and elevates intracellular cathepsin K levels but impairs bone resorption. Conversely, overexpression of wild-type *Rufy4* promotes actin assembly and active bone resorption while simultaneously inhibiting differentiation. Meanwhile, overexpression of *Rufy4* lacking the RUN domain promotes osteoclastogenesis, elevates intracellular cathepsin K levels, and inhibits bone resorption. These results underscore *Rufy4*’s pivotal role in osteoclast differentiation and function through its regulation of cytoskeletal dynamics, suggesting its potential as a therapeutic target for osteolytic diseases caused by osteoclast hyperfunction. In this study, the intricate relationship between *Rufy4* and oxidative stress remains incompletely elucidated. However, the expression of *Rufy4* in osteoclasts, which undergo differentiation in response to oxidative stress, suggests its potential role in bone resorption. Interestingly, sulforaphane, an Nrf2-activating phytochemical, reduces oxidative stress. Previously, we found that sulforaphane downregulates *Rufy4* mRNA expression and inhibits bone resorption in osteoclasts (unpublished data). Although further experiments are required to understand the underlying mechanisms, targeting Rufy4 expression holds promise for developing new medicines aimed at inhibiting bone resorption.

## Data Availability

The original contributions presented in the study are included in the article/Supplementary Material. Further inquiries can be directed to the corresponding author (E.S.). The microarray datasets mentioned in this article are not readily available as they are part of an ongoing study. Requests to access the datasets should be directed to the corresponding author (E.S.).

## Supplementary Materials

The following supporting information can be downloaded at: https://www.mdpi.com/article/10.3390/cells13211766/s1, Figure S1: Overexpression of FLAG-tagged wild-type RUFY4 inhibits osteoclast formation; Figure S2: FLAG-tagged wild-type RUFY4 colocalizes LAMP2; Figure S3: The midbody marker CRIK indicates that the intercellular bridge is in a cytokinesis state; Video S1: Control RAW-D cells were cultured on glass-bottom dishes in α-MEM supplemented with 10% FBS and 100 ng/mL RANKL for 48 h. Time-lapse images were captured at 6-min intervals for 12 h; Video S2: RAW-D cells overexpressing FLAG-tagged wild-type RUFY4 were cultured on glass-bottom dishes in α-MEM supplemented with 10% FBS and 100 ng/mL RANKL for 48 h. Time-lapse images were then captured at 6-min intervals for 12 h; Video S3: RAW-D cells overexpressing FLAG-tagged RUFY4 RUN domain-deletion mutant were cultured on glass-bottom dishes in α-MEM supplemented with 10% FBS and 100 ng/mL RANKL for 48 h. Time-lapse images were then captured at 6-min intervals for 12 h; Figure S4: Raw western blotting images corresponding to Figure 2b, Figure 4b,g, and Figure 5b,h. The markers could not be visualized in the raw membrane data because three-colored pre-stained markers without chemiluminescent substances were used.
