# Supplementary material for: Autophagy Regulator Rufy 4 Promotes Osteoclastic Bone Resorption by Orchestrating Cytoskeletal Organization via Its RUN Domain
Source: Cells. 2024 Oct 25;13(21):1766. doi: 10.3390/cells13211766 (PMC11545195; doi:10.3390/cells13211766)
Supplement: Supplementary file 1 [file cells-13-01766-s001.zip › Supple Figure.pdf]

Supplementary Materials

Autophagy regulator Rufy4 promotes osteoclastic bone resorption by orchestrating cytoskeletal organization via its RUN domain

Eiko Sakai, Minoru Saito, Yu Koyanagi, Yoshitsugu Takayama, Fatima Farhana, Yu Yamaguchi and Takayuki Tsukuba

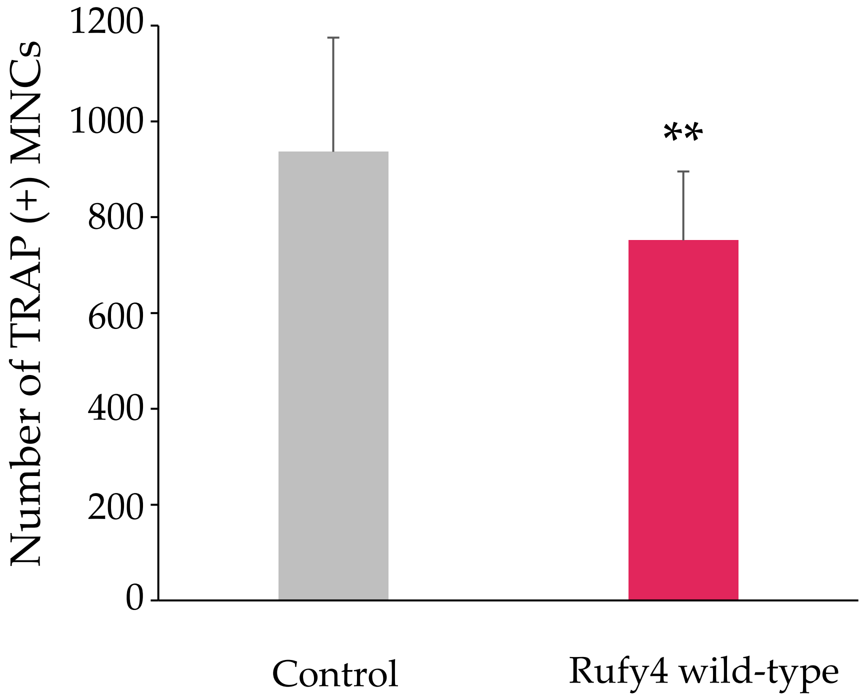

Supplementary Figure S1:
Overexpression of FLAG-tagged wild-type RUFY4 inhibits osteoclast formation

Control (empty vector) or FLAG-tagged Rufy4 wild-type-overexpressing RAW-D cells were stimulated with 100 ng/mL RANKL for three days. The cells were then fixed and stained for TRAP. Number of TRAP-positive multinucleated cells was counted. Data are presented as the mean ± SD from three independent experiments. The significance of differences was assessed using Student's *t*-test (***P*<0.01).

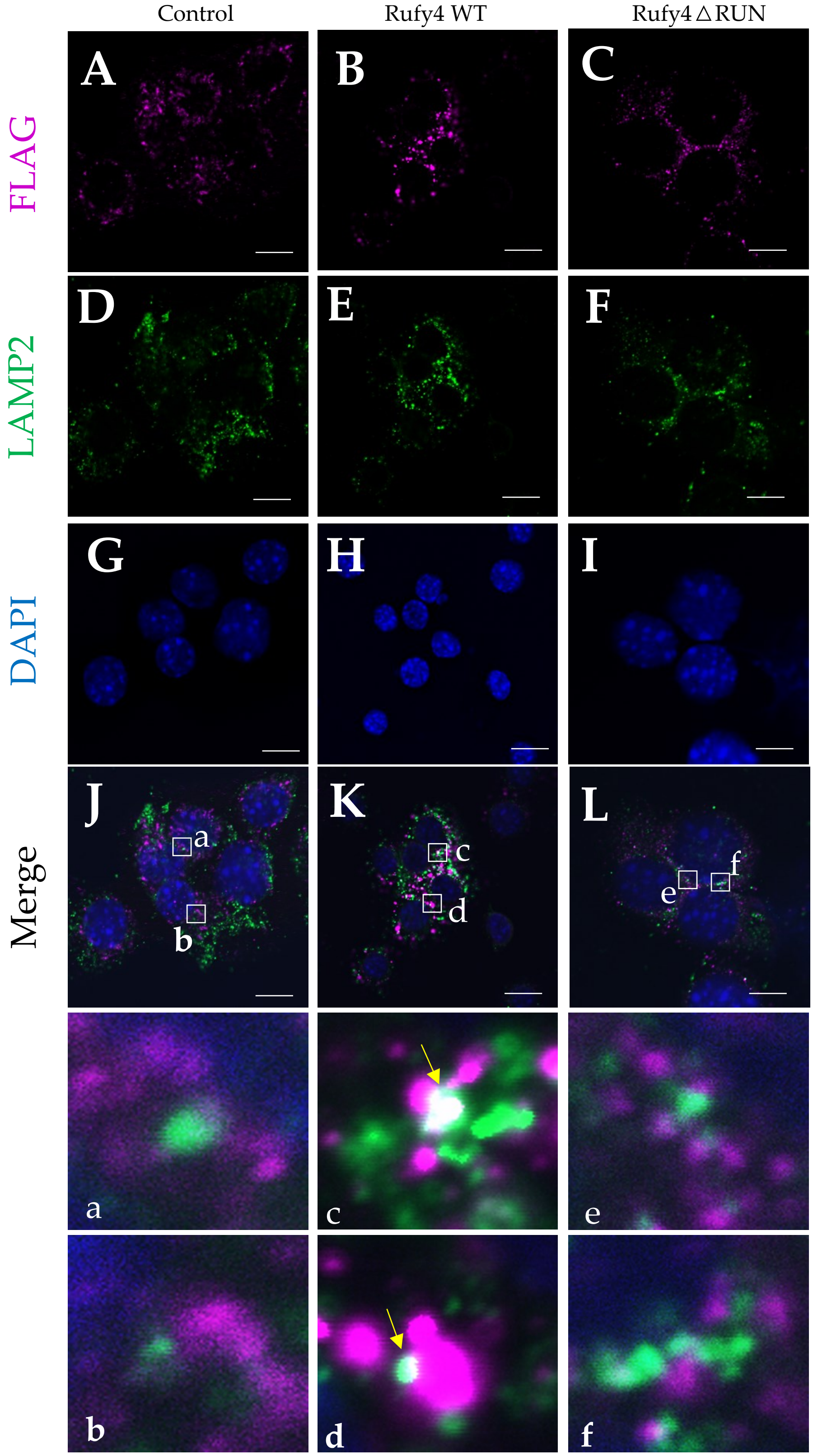

Supplementary Figure S2

RAW-D cells overexpressing the control (A, D, G, and J), FLAG-tagged Rufy4 wild-type (B, E, H, and K), and FLAG-tagged Rufy4 RUN-domain deletion mutant (C, F, I, and L) were cultured with 100 ng/mL RANKL. After 3 days, cells washed with PBS and fixed, stained with anti-FLAG antibody, anti-LAMP2 antibody, and DAPI, and visualized using confocal microscopy. (a, b) High-magnification image of square a and b in panel J. (c, d) High-magnification image of square c and d in panel K. (e, f) High-magnification image of square e and f in panel L. Colocalization of FLAG and LAMP2 were observed in Rufy4 overexpressing osteoclasts (c, d, arrows). Scale bar, 20 μm.

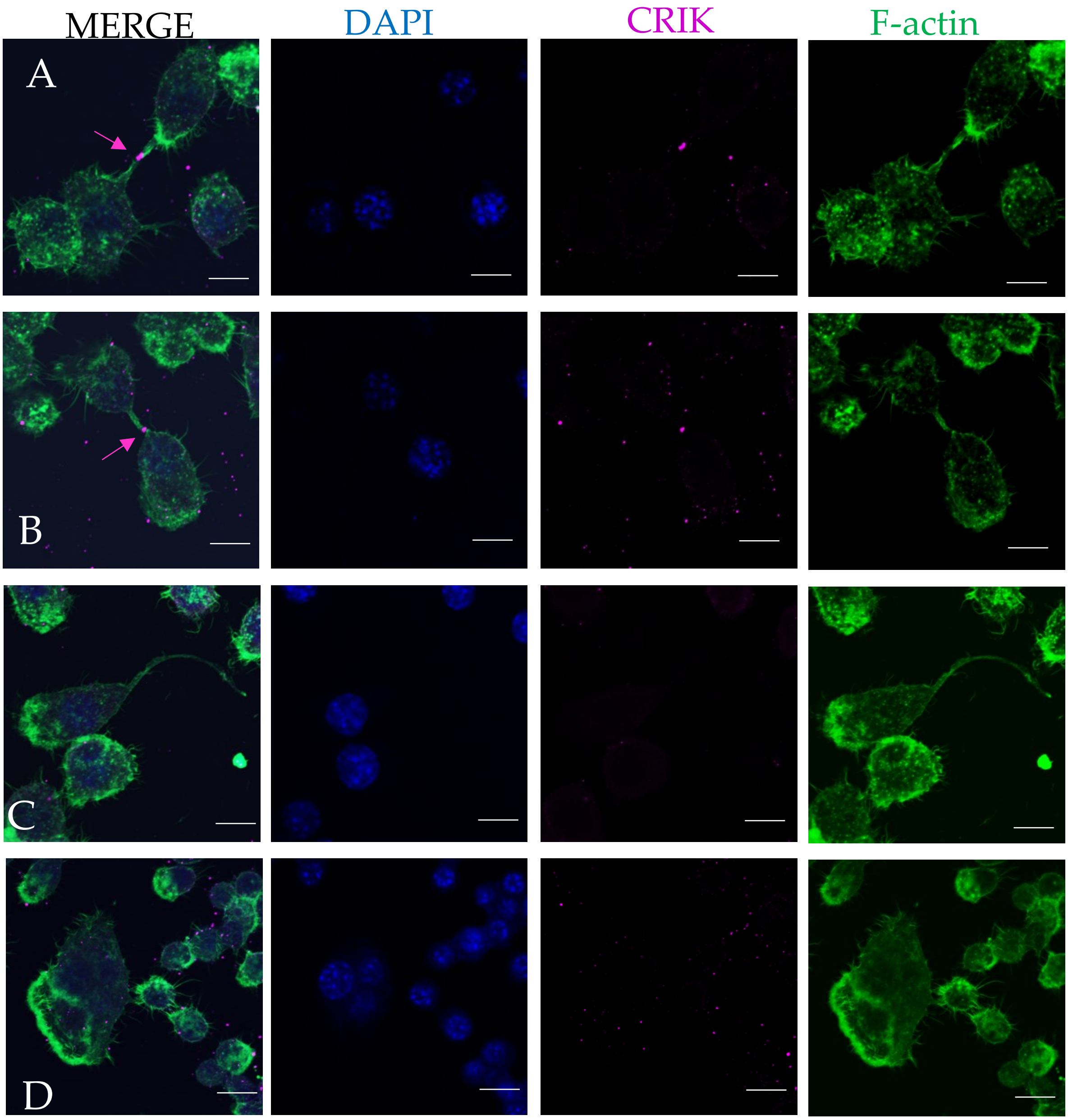

Supplementary Figure S3

RUN domain lacking Rufy4 overexpressed cells were stimulated with RANKL.
After 3 days, cells washed with PBS and fixed, then stained with Alexa Fluor 488 phalloidin, anti-CRIK antibody, and DAPI. Midbody marker CRIK was observed in cytokinetic bridge (A and B, arrows). Scale bar, 20 μm.

Figure 2 (b)

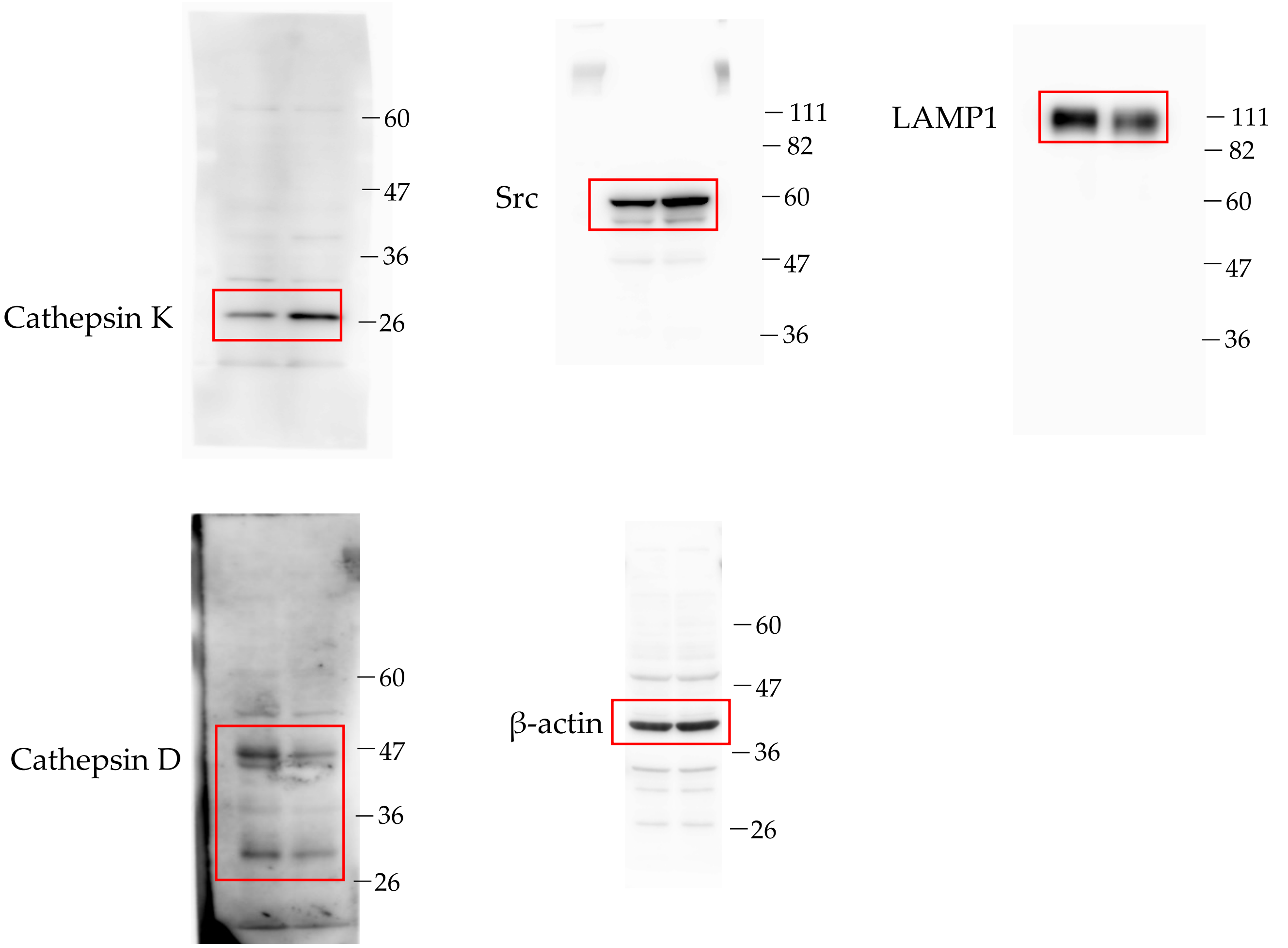

Figure 4 (b)

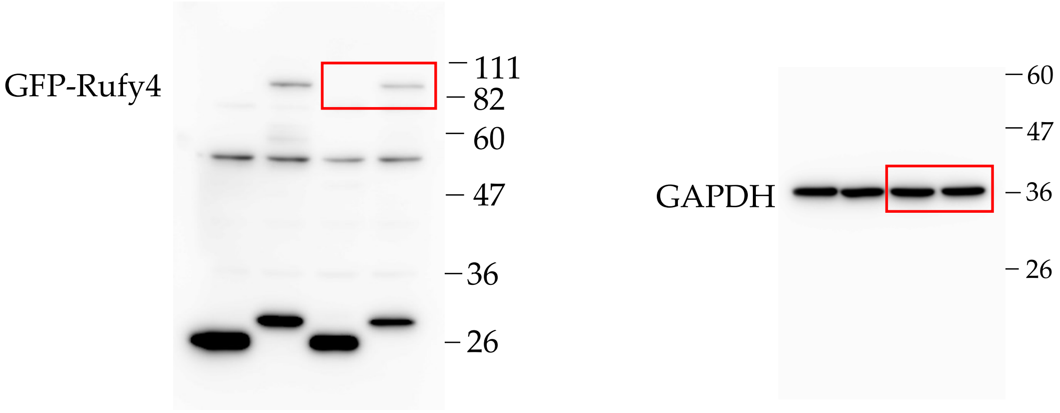

Supplementary Figure S4

Figure 4 (g)

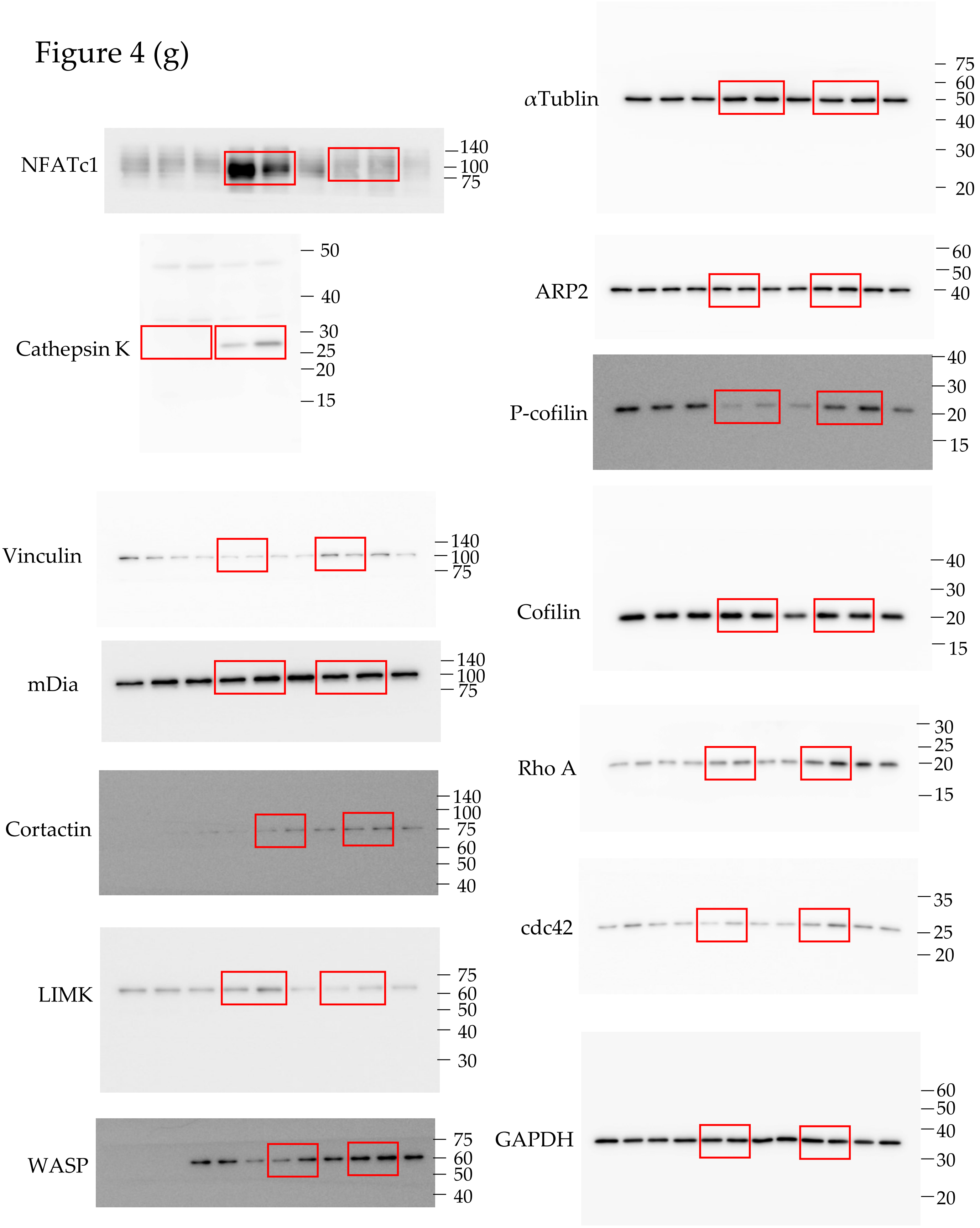

Supplementary Figure S4

Fig. 5 (b)

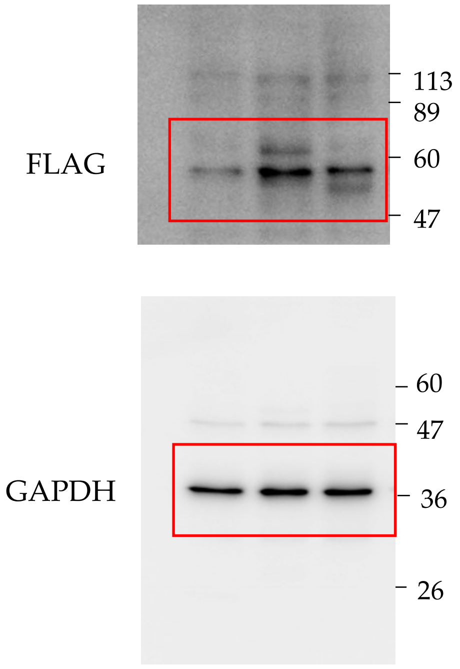

Fig. 5 (h)

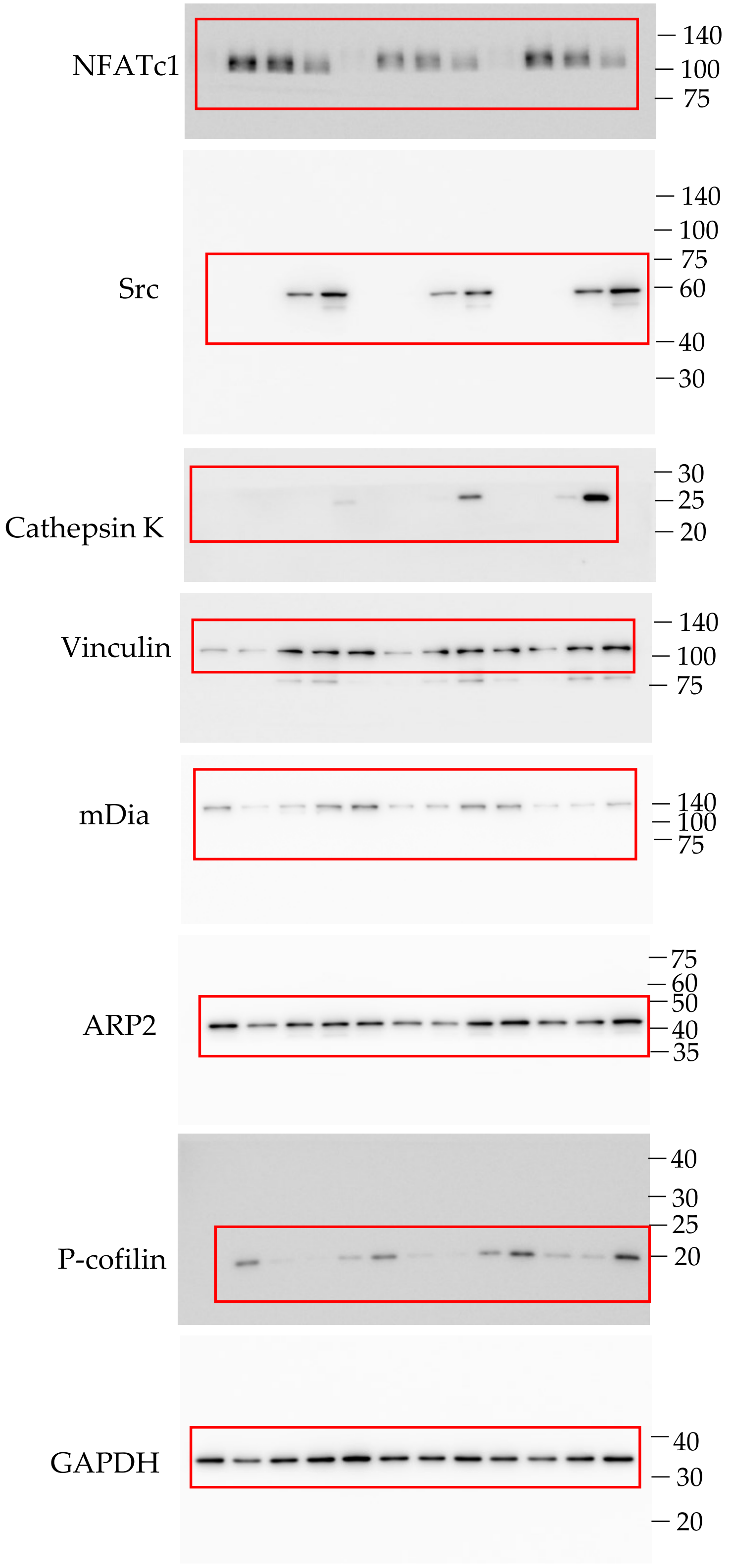

Fig. 6 (f)

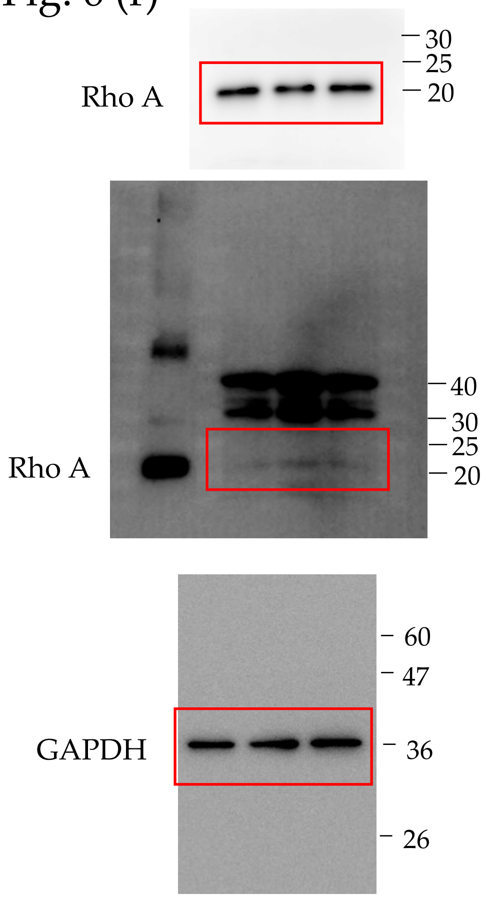

Supplementary Figure S4

Fig. 6 (g)

Talin

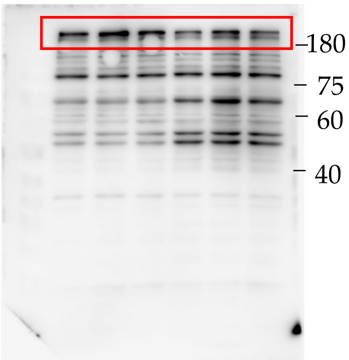

Cortactin

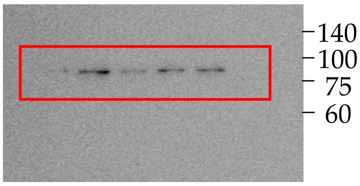

WASP

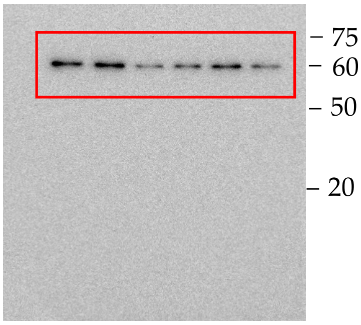

LIMK

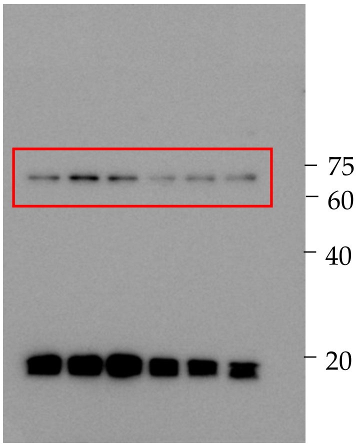

GAPDH

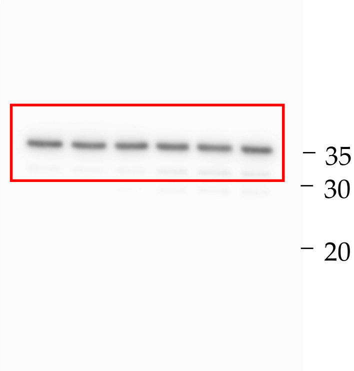

Supplementary Figure S4
